# Sex-specific expression of circadian rhythms enables allochronic speciation

**DOI:** 10.1093/evlett/qrae049

**Published:** 2024-10-08

**Authors:** G Sander van Doorn, Jens Schepers, Roelof A Hut, Astrid T Groot

**Affiliations:** Groningen Institute for Evolutionary Life Sciences, University of Groningen, Faculty of Science and Engineering, The Netherlands; Groningen Institute for Evolutionary Life Sciences, University of Groningen, Faculty of Science and Engineering, The Netherlands; Groningen Institute for Evolutionary Life Sciences, University of Groningen, Faculty of Science and Engineering, The Netherlands; Institute for Biodiversity and Ecosystem Dynamics, University of Amsterdam, Faculty of Science, The Netherlands

**Keywords:** allochronic diversification, phenotype matching, circadian rhythm, Spodoptera frugiperda, intra-locus sexual conflict, sex-specific gene expression

## Abstract

Noctuid moths provide prime examples of species in various stages of allochronic speciation, where reproductive barriers are mediated by genetic divergence in daily or seasonal timing. Theory indicates that allochronic divergence might be one of the most plausible mechanisms of adaptive speciation, especially when timing is subject to divergent ecological selection. Here, we show that the validity of this theoretical expectation is entirely contingent on species characteristics of the mating system. Our analysis focuses on the moth *Spodoptera frugiperda* (Lepidoptera, Noctuidae), which occurs as two strains that differ in circadian reproductive activity. Unlike in generic models of assortative mating, where chronotypes diverge under mild assumptions, individual-based evolutionary simulations of the mating system and life cycle of *S. frugiperda* fail to recover allochronic diversification, even under conditions highly conducive to speciation. Instead, we observe that both chronotypes advance their activity schedule toward the early night, resulting in a rapid loss of allochronic variation. This outcome is caused by the fact that mating in *S. frugiperda* takes considerable time and potential mates are encountered sequentially, so that early males enjoy a systematic advantage. The undermining effect of male mate competition can be overcome when circadian genes evolve sex-specific expression, enabling early and late chronotypes to be maintained or even to diversify in sympatry. These results give new significance to sex differences in biological rhythms and suggest that species characteristics of the mating system and genetic architecture are key to understanding the scope for allochronic speciation across diverse species exhibiting variation in timing.

## Introduction

The origin of biodiversity and speciation are long-standing topics of interest in evolutionary biology. Classical speciation theory and the more recent development of models for adaptive diversification have provided a solid understanding of the fundamental processes that are involved in speciation with and without gene flow (Kirkpatrick & Ravigné, 2004; Coyne & Orr, 2004; Dieckmann et al., 2004; Gavrilets, 2004; Weissing et al., 2011). Yet, it remains a challenge to connect this body of theory with empirical data. A major complicating factor is that the evolution of reproductive isolation and ecological diversification in natural populations are influenced by many processes that interact with main drivers of speciation but that are largely ignored in theoretical models for the sake of parsimony. Moreover, speciation facilitated by multi-effect traits (Servedio et al., 2011) and the growing availability of data on the genomic basis of speciation traits (Wolf & Ellegren, 2017; Campbell et al., 2018) suggest that genetic architecture (e.g., pleiotropy or patterns of physical genelinkage) might be as important as the configuration of selective forces in determining speciation. This attributes much greater explanatory weight to species-specific details than is accounted for in the generic predictions of conceptual speciation models.

Models tailored to specific case studies (e.g., Gavrilets et al., 2007; Gavrilets & Vose, 2007) provide a helpful stepping stone to bridge the current gap between theory and empirical research. Well-chosen case studies not only offer detailed insight into the particular system under consideration; they also help integrate existing conceptual models, showcasing how each can serve as an explanatory building block for resolving the interplay of factors contributing to natural speciation processes. Here, we apply this approach to study allochronic diversification in the fall armyworm moth, *Spodoptera frugiperda*. *Spodoptera frugiperda* is a noctuid moth (Lepidoptera: Noctuidae) native to South- and North-America, which recently invaded into Africa and South-East Asia (Goergen et al., 2016; Hruska, 2019). As the larvae feed on a wide range of host plants and are capable of inflicting extensive damage on a range of important crops, *S. frugiperda* is regarded as a major agricultural pest and has been studied in depth (Groot et al., 2010; Bateman et al., 2018). Small differences in the biology of the species appear to exist between South American, North American and invasive populations (Saldamando Benjumea et al., 2014; Yainna et al., 2022; Durand et al., 2024); here we focus on data collected from North America.

Early genetic work by Pashley, (1986) revealed that North American fall armyworm occurs as two strains that are morphologically indistinguishable but genetically differentiated. The two strains show overlap in host plant utilization, although one (the corn strain) tends to be found more often on tall grasses (e.g., corn, sorghum, cotton), whereas the other (the rice strain) occurs predominantly on rice and small pasture grasses (e.g., bermudagrass) (Pashley, 1986, 1988; Prowell et al., 2004; Meagher et al., 2004). Therefore, it has been suggested that *S. frugiperda* is an example of a generalist herbivorous insect species that is undergoing sympatric speciation through habitat isolation (Pashley et al., 1995; Dres & Mallet, 2002; Fiteni et al., 2022; Nam et al., 2024). However, field observations and lab studies on oviposition preference, larval feeding preference and larval performance showed only weak host-plant association (Juarez et al., 2019). Several other mechanisms have been proposed to account for the separation of the two strains (Groot et al., 2010), including postzygotic isolation due to reduced fertility of hybrid females (Kost et al., 2016), behavioural isolation mediated by divergence in sex-pheromone signals (Unbehend et al., 2013), and isolation caused by difference in timing of reproductive activity during the night (Pashley et al., 1992). In fact, although temporal differentiation is not observed universally across the species’ range (e.g., Saldamando Benjumea et al., 2014), it was repeatedly reported as the predominant phenotypic difference between the corn and rice strain, both infield-studies (Pashley et al., 1992; Schöfl et al., 2009, 2011) and lab-reared populations (Hänniger et al., 2017). This led to the suggestion that gene flow between the two sub-populations may be limited primarily by the existence of alternative chronotypes (Groot et al., 2008; Tessnow et al., 2022), similar to allochronic strains in other moth species that exhibit variation in circadian (Groot, 2014) or seasonal timing (e.g., the European corn borer *Ostrinia nubilalis*, Dopman et al., 2010; the pine processionary moth *Thaumetopoea pityocampa*, Santos et al., 2011; or the Japanese geometrid moth *Inurois punctigera*, Yamamoto & Sota, 2009).

Divergence in the timing of reproductive activity generates, as an automatic by-product, a bias toward matings between partners with overlapping activity profiles. This is thus a typical case of phenotype matching (Kopp et al., 2018), or similarity-based mating (Gavrilets, 2004), equivalent to mechanisms of positive assortative mating that are frequently assumed in models of ecological speciation (e.g., Dieckmann & Doebeli, 1999; Matessi et al., 2001; Bürger et al., 2006; Gavrilets & Vose, 2007; Rettelbach et al., 2013). These generic, conceptual models show that matching rules for mating create ideal conditions for speciation, because they require only a single trait to diversify in order to establish prezygotic reproductive isolation (Kopp et al., 2018). Therefore, the theoretical literature motivates the prior expectation that speciation in *S. frugiperda* is a relatively straightforward process: the divergence of only circadian timing would be sufficient to generate assortative mating and reduce gene flow between sub-populations of different chronotypes, making it easier for them to specialize into different ecological niches and establish stable coexistence. Moreover, if timing were under direct divergent ecological selection as well (i.e., if it were a multiple-effect trait; Servedio et al., 2011), speciation would potentially occur very rapidly, as the evolution of assortative mating and ecological specialisation to host-plant habitats would then go hand-in-hand (Fry, 2003; Smadja & Butlin, 2011).

Here, we test these intuitions by developing an evolutionary model of the life cycle of *S. frugiperda* and show that allochronic divergence between corn and rice strains is undermined by a conflict between males and females over the optimal timing of activity, a source of selection that is ignored by generic phenotype-matching models of speciation. We identify the root cause of this (intralocus) sexual conflict in the mating system of *S. frugiperda* and demonstrate that the resolution of conflict, by means of the evolution of sex-specific differences in timing, is essential for allochronic divergence to occur. Our analysis underscores the importance of mechanistic details of both the mating system and the genetic architecture of speciation traits for explaining diversification patterns in natural populations while nuancing the broad categorization of speciation mechanisms suggested by conceptual theory (Kirkpatrick & Ravigné, 2004; Weissing et al., 2011; Smadja & Butlin, 2011; Kopp et al., 2018).

## Results

The evolutionary model was set up as an individual-based simulation that followed a population of individuals through a life-cycle mimicking the biology of (North American) *S. frugiperda* (see “Methods” section); its basic assumptions were comparable to those of earlier ecological speciation models with phenotype matching as a mechanism for assortative mating (Supplementary Analysis).

In *S. frugiperda*, receptive females are active for a limited period of time during the night, when they may attract multiple males that overlap in activity. Once a female accepts a male as mating partner, copulation extends over several hours (e.g., Schöfl et al., 2009), preventing both partners from securing another mate before the break of day. In the model, these mating characteristics were captured by assuming that encounters between potential mates were determined exclusively by circadian timing, a genetically encoded trait (denoted as τ) that controlled an individual’s profile of activity during the night (Floessner & Hut, 2017); values of τ are expressed on a continuous scale, ranging from 0 (beginning of the night) to 1 (end of the night). Individuals were more likely to encounter potential partners that overlapped in activity. Moreover, we assumed that individuals were available on the mating market only for as long as they had not yet mated during the same night. Males could mate every night, whereas females were not available anymore after they had mated.

The mating interactions were simulated in the context of the full life-cycle of *S. frugiperda*: after adult females had selected a mate from the pool of locally available males, they deposited their eggs in either the local corn or rice habitat; the larvae subsequently developed on their host plant and were exposed to density-dependent competition within their local subpopulation (deme); surviving juveniles then replaced the parental generation and were able to migrate to other demes before producing the next generation of offspring. Mating success and survival were affected by the timing trait τ, and the gene loci that encoded this trait were subject to mutation and transmitted from parent to offspring following the rules of Mendelian genetics for diploid, unlinked autosomal genes. By simulating this process over the course of many generations, we were able to study the evolution of the population and evaluate its tendency to undergo allochronic diversification.

### A rapid loss of polymorphism interferes with allochronic diversification

**Figure 1:**
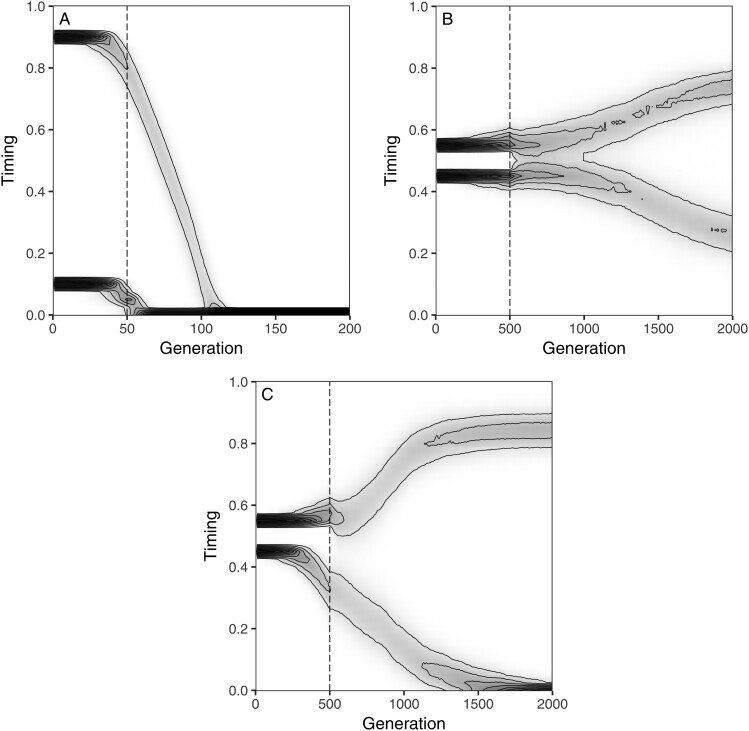
Divergence of circadian rhythms depends on the mating system. Plots show the evolution of the phenotypic distribution of the timing trait τ through time for three versions of the model: (A) baseline model representing the mating system of *S. frugiperda* (with asymmetric mate competition due to first-male precedence); (B) alternative mating system with male scramble competition for access to females; (C) monogamous mating system. All three simulations implement a secondary-contact scenario with either high (A) or low (B and C) initial divergence; dashed lines mark the transition between a period of allopatry and a subsequent phase of restored gene flow between sub-populations. Throughout, timing is subject to diversifying ecological selection, mediated by differential juvenile survival in the corn vs. rice habitat. Model details and parameter conditions are specified in “Methods” section.

Simulations of the baseline version of the model show that genetic variation in circadian timing is lost rapidly from the population, even under conditions highly conducive to adaptive diversification, as in Figure 1A. Here, we simulated a secondary contact scenario between populations that exhibited large initial differences in circadian timing, and we assumed that timing was subject to divergent ecological selection between the corn and the rice habitat (i.e., τ was considered to be a multiple-effect trait). A comparable generic model that incorporates phenotype matching as a basis for assortative mating readily allows for ecological speciation under these conditions (Supplementary Analysis). Accordingly, we inferred that a particular detail of the mating system of *S. frugiperda* must be responsible for the observed loss of variation in circadian timing.

Because initial differences in timing already start to erode rapidly in isolated populations before secondary contact is established (indicated by the dashed vertical line in Figure 1A), we argued that variation is lost due to a source of directional selection that drives the activity patterns of individuals toward early in the night (τ→0). We hypothesized that this source of selection acted on males: due to the fact that mating in *S. frugiperda* takes substantial time, individuals already engaged in mating will not be available as partner for others during the same night; therefore, individuals who become active late run a significant risk of remaining unmated (Hau et al., 2017), and this is particularly costly for males since their reproductive output is proportional to mating success. By contrast, the reproductive output of a female is limited by the amount of resources she can allocate towards reproduction; her reproductive success will suffer from a low mating rate only if she fails to attract any mate over the duration of the mating season.

### Asymmetric male competition causes directional sexual selection but not in alternative mating systems

To test the hypothesis that males are under selection to become active early in order to maximize their access to mating partners and that this source of directional selection is strong enough to obstruct diversification, we implemented two alternative mating systems and studied their effect on the evolution of circadian timing. Both mating systems were not intended to reflect the actual mating system of *S. frugiperda*, but a hypothetical variant of it in which one or multiple sources of sexual selection on males had been eliminated.

The first alternative mating system emulated a generic phenotype matching mechanism characteristic of existing models of ecological speciation. In this system, we assumed (a) that mating interactions took negligible time so that individuals could mate arbitrarily often in a given night, and (b) that mating tended to occur between individuals who were active at the same time. After each night, mated females produced a single clutch of eggs, fertilized by sperm that the female collected during that night, where all of her mates had the same probability of fertilising the eggs. The second alternative mating system was a monogamous mating system. Here, mate encounters were modelled in the same way as in the baseline model (i.e., sequentially, with an extended time of mating), except that males as well as females were assumed to mate only once during the entire mating season. As a result, males disappeared from the mating market at the same rate as females, causing the operational sex ratio to remain constant and equal on average.

As shown in Figure 1B and C, both alternative mating systems profoundly influenced the regime of competition for access to mates experienced by males, while they only required minor adjustments to the model implementation. In the first alternative mating system, the competitive regime between males takes the form of (symmetric) scramble competition. Unlike in the simulation of the mating system of *S. frugiperda* (Figure 1A), where male–male competition was asymmetric due to first-male precedence, symmetric scramble competition (Figure 1B) generates no directional sexual selection, so that the population can undergo adaptive diversification, even when initial allochronic differentiation is low. In this variant of the model, divergence builds up slowly because phenotype matching still acts as a source of stabilizing selection on male timing (Kirkpatrick & Nuismer, 2004). Males with intermediate timing initially benefit from being able to mate with a larger fraction of the female population, delaying the early phase of diversification shortly after the establishment of secondary contact. Diversification is more rapid and pronounced when mating is monogamous, as in the second alternative mating system (Figure 1C). Here, monogamy suppresses variation in male reproductive success, largely eliminating the basis for sexual selection. As a result, circadian timing evolves in response to predominantly ecologically divergent selection.

In summary, the two alternative models support our hypothesis that males are under selection to shift their activity schedule toward the early night and clarify that divergent selection on timing is overpowered by directional sexual selection, induced by sequential access to mates, prolonged mating and sex differences in potential reproductive rate (Hau et al., 2017). Moreover, since neither of the alternative mating systems are consistent with the biology of the study species and a rapid loss of allochronic variation is observed across a broad range of parameters for the baseline model (Supplementary Figure S1A), we come to the conclusion that the model, so far, fails to explain why differences in timing are maintained between the corn and rice strain of *S. frugiperda*.

To address this problem, we asked whether additional reproductive barriers, such as habitat choice or the limited activity of hybrid females, could interact with timing to rescue allochronic diversification in the face of directional sexual selection. However, the results of these simulations were negative and we could never identify a reasonable range of parameter conditions where divergence would occur or be maintained (SupplementaryFigure S2).

### Sex-differential expression of timing genes is required for allochronic diversification

Given that the outcome of the model changed qualitatively when we altered the mating system to manipulate the mechanism of male–male competition (Figure 1A vs. 1B and C; Supplementary Figure S1), we reasoned that timing might be subject to different selection regimes in males and females, creating conditions for intralocus conflict between the sexes (Bonduriansky & Chenoweth, 2009; van Doorn, 2009). This type of sexual conflict is characterized by an evolutionary tug-of-war in which the optimization of the trait in males is associated with sub-optimal trait expression in females and vice-versa. Intra-locus sexual conflict can be resolved by differential gene expression in the two sexes (Ellegren & Parsch, 2007), so we decided to test whether our simulation would yield different results if we allowed for the evolution of sex-specific expression of circadian timing.

**Figure 2:**
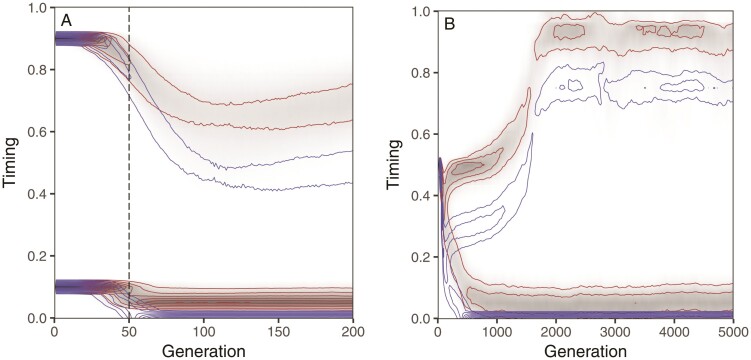
The resolution of sexual conflict over timing leads the way to allochronic diversification. Two individual-based simulations showing the maintenance (A) and emergence in sympatry (B) of two reproductively isolated sub-populations that differ in daily timing of mating. Simulations replicate the conditions of Figure 1A, except that timing genes were enabled to evolve a sex-specific pattern of expression (see “Methods” section), creating opportunity for the resolution of intra-locus sexual conflict. As a result, the distribution of female (red contours) and juvenile timing (grey shading) maintains a degree of local adaptation to the corn and rice habitat, forcing males (blue contours) to evolve a compromise between advancing their activity schedule and maintaining an overlap in activity with females of their own chronotype.

When the evolution of sex-differential expression of timing genes was enabled in the simulations (see “Methods” section), the initial difference in timing between the corn and rice strain persisted, maintaining strong reproductive isolation between the two sub-populations (Figure 2A). Sex-differential gene expression did not prevent males from shifting their activity profile toward early night. However, it allowed females and juveniles of the rice strain to maintain an activity profile closer to the end of night, which is the optimal timing for juvenile survival in the rice habitat in our model. As a consequence, rice-strain males were forced to find a compromise between the competitive advantage of being active before other males and the need to overlap in activity with the (locally abundant) rice-strain females, which prevented them from shifting their activity too far ahead of the females of their own strain. Also the corn-strain males maintain an activity schedule that is a bit earlier than that of corn-strain females and juveniles. This is a result of mutation bias and unequal strengths of selection between sexes, which allows for some standing genetic variation in timing to accumulate in females and juveniles, whereas mutations in male timing are more effectively purged by strong selection.

Interestingly, the resolution of sexual conflict can remove obstacles to allochronic diversification also in a sympatric biogeographical setting, as in the simulation in Figure 2B, which was started with no initial genetic variation in timing and without an initial phase of geographic isolation. This particular simulation was run for a longer period of time (5,000 generations) to demonstrate that the evolved polymorphism is stable; in fact, the transient dynamics visible in Figure 2A converges on the same stable state if this simulation is extended to also run for 5,000 generations (data not shown).

**Figure 3:**
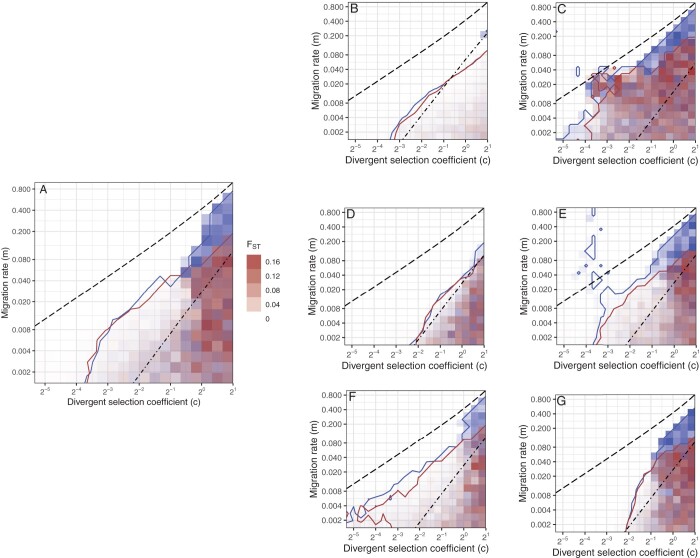
Evolution of reproductive isolation for a range of bio-geographic conditions and divergent selection regimes. Simulations incorporating sex-specific expression of timing were run for 20 × 20 combinations of c and m, two key model parameters that determine, respectively, the strength of divergent ecological selection on timing and the rate of migration between demes. Two sets of simulations are represented in each panel: one reflecting a secondary-contact scenario (blue), the other divergence in sympatry (red). Each simulation was run for 10,000 generations, at which point we measured the correlation in chronotype between mating partners (red and blue contour lines indicate $r_{\mathrm{MF}}=0.8$) and $F_{\mathrm{ST}}$ (background colour) between early and late sub-populations, based on divergence at a set of 20 neutral diploid loci. Both measures of reproductive isolation relied on a classification of individuals in sub-populations of early and late chronotypes, which we determined by a k-means clustering algorithm (Loyd, 1982). The dashed and dot-dashed black lines delineate qualitatively different selective regimes across the parameter space (see text and Supplementary Analysis). Main panel: default parameter set (see “Methods” section); (B and C) wider (a=20.) vs. narrower (a=100.) profile of activity; (D and E) lower (η=5.) vs. higher (η=100.) rate of encounters; (F and G) lower ($s_{r}=0.0$) vs. higher ($s_{r}=0.2$) cost of sex-specific gene regulation.

Additional simulations, in which we systematically varied the strength of ecological divergent selection and the rate of migration between subpopulations, show that sex-specific allochronic diversification occurs under a broad range of conditions, largely irrespective of the level of initial divergence (Figure 3; blue versus red contours/shading, respectively, reflect simulations of a secondary-contact scenario versus a sympatric biogeographic setting).

The parameter space in Figure 3 is separated into three regions by black lines that were derived from analytical conditions for the evolutionary branching of circadian timing in a generic model of assortative mating (Supplementary Analysis) and that serve as benchmarks for interpreting the simulation results: the dashed line separates between conditions where ecological selection is stabilizing (to the left and above) versus disruptive (to the right and below); the dot-dashed line does the same for the net effect of the combination of ecological selection and assortative mating (which, under conditions of sympatry with limited genetic variation, acts as a source of stabilizing selection on males; (Kirkpatrick & Nuismer, 2004)). Accordingly, net selection is stabilizing on both sexes in the upper-left region and disruptive on average across males and females in the lower-right region of the parameter space; in the middle region between the two lines, net selection is stabilizing at the population level but qualitatively different between the sexes; that is, stabilizing in males but disruptive in females.

Allochronic diversification, which was indicated by strong premating isolation ($r_{\mathrm{MF}}>0.8$; to the right and below the red/blue contour lines), is observed almost always when the combined effect of ecological and sexual selection on timing is disruptive on average across both sexes (Figure 3), confirming that the evolution of sex-specific timing removes the problem of directional sexual selection on males across a broad range of ecological parameters and biogeographical settings. Interestingly, divergence in sympatry is also observed for some parameter combinations in the region between the dashed and dot-dashed reference lines, where the net force of selection on the population is computed to be stabilising in the absence of sex-limited expression (Supplementary Analysis). This would seem to suggest that sex-specific timing also diminishes the stabilizing effect of assortative mating on males that has been observed in previous models of phenotype matching (Kirkpatrick & Nuismer, 2004). However, incorporating sex-limited expression in simulations with male scramble competition or a monogamous system does not relax the conditions for allochronic diversification (Supplementary Figure S3). Furthermore, several factors besides the balance between selective forces are observed to co-determine the feasibility of diversification in the intermediate region of the parameter space, where the sexes are subject to qualitatively different regimes of selection, including biogeographical conditions, the rate of mate encounters and the specificity of nonrandom mating (Figure 3B–E), and the costs of sex-specific gene expression (Figure 3F and G).

Strong premating isolation (high $r_{\mathrm{MF}}$) was frequently accompanied by strong genetic differentiation (measured as high $F_{\mathrm{ST}}$ values computed for a set of neutral marker loci), indicating that the population is differentiated into two genetically isolated sub-populations. However, premating isolation did not always coincide with elevated $F_{\mathrm{ST}}$. This occurs, for example, in Figure 3B, where the broad activity profiles of individuals allowed for frequent exchange of neutral gene variants between chronotypes, so that $F_{\mathrm{ST}}$ always remained low, despite the maintenance of variation in timing across the lower right area of the parameter space. In these and other simulations with high $r_{\mathrm{MF}}$ but low $F_{\mathrm{ST}}$, we observed the maintenance of a broad distribution of timing in the population but no separation of the gene pool in discrete clusters.

## Discussion

### Implications for speciation theory

Phenotype matching occurs when choosy individuals select a mate on the basis of a match with their own phenotype. As a mechanism for positive assortative mating, it is far less widespread than mate choice mediated by separate preferences and display traits that have diverged between species (Hauber & Sherman, 2001). Nevertheless, phenotype matching is the default assumption in the majority of theoretical models of ecological speciation (e.g., Dieckmann & Doebeli, 1999; Matessi et al., 2001; Bürger et al., 2006; Gavrilets & Vose, 2007; Rettelbach et al., 2013), (but see Doebeli, 2005; Gavrilets et al., 2007, for exceptions). The reason is that conditions for speciation tend to be less restrictive in models with phenotype matching, where reproductive isolation can emerge when only the cue for self-referent matching diverges between the daughter species (Smadja & Butlin, 2011; Kopp et al., 2018). By contrast, positive assortative mating based on separate preferences and mating signals relies on the divergence of both traits and on the maintenance of positive linkage disequilibrium between them, despite recombination (Felsenstein, 1981).

The limited biological examples of phenotype matching encompass several mechanisms in diverse phylogenetic groups, such as song imprinting in birds (ten Cate & Vos, 1999), preferences for host plants in phytophagous insects that mate on their host (Fry, 2003), or allochrony, which has driven divergence in multiple groups of organisms (Taylor & Friesen, 2017), including acroporid corals, plants with differentiated flowering phenology, and moths with intraspecific variation in activity profiles (Groot, 2014; Dopman et al., 2010; Santos et al., 2011; Yamamoto & Sota, 2009). We here modelled an example of the latter category as a case-study of allochronic diversification that integrated insights from generic models of ecological speciation with specific knowledge of the biology of *S. frugiperda*.

To our initial surprise, and in contrast to the general assumption that speciation based on a phenotype-matching mechanism should be easy, our analysis revealed that allochronic speciation is obstructed when mates are encountered sequentially and mating takes considerable time, as is the case in *S. frugiperda* (Schöfl et al., 2009). Also in combination with additional reproductive barriers, preexisting allochronic differentiation is rapidly eroded due to strong sexual selection on males (Supplementary Figure S2; Hau et al., 2017), who can maximize their access to mating partners by becoming active earlier during the night. This effect remained unnoticed in generic models of ecological speciation because their treatment of phenotype matching focused on the outcome of assortative mating without explicitly specifying the process by which individuals select their mate. While such models appear to be general, their predictions may, in fact, be vulnerable to seemingly trivial details of the mechanisms of mate choice. Individual-based simulation proves to be a helpful tool to uncover such vulnerabilities: unlike analytical approaches, they force the modeller to provide an explicit description of the mate choice process, which can subsequently be altered relatively straightforwardly to explore the consequences of alternative assumptions (see, e.g., Figure 1). In this way, generic models and case studies, or analytical and simulation methods, can function as complementary tools in a pluralistic modelling approach.

### The resolution of sexual conflict as a driver of speciation

The realization that sex role asymmetry leads to a different balance of disruptive, stabilizing and directional selection forces between males and females motivated us to explore the potential of sex-specific gene regulation to resolve sexual conflict over timing. Sex-specific expression of circadian genes enabled females to adapt divergently to the corn and rice habitat, so that the rice-strain males could no longer advance their schedule without compromising their overlap in activity with the rice-strain females. Unlike any of the subsidiary reproductive isolation mechanisms that we considered, the evolution of sex-specific timing was able to rescue allochronic diversification over a broad range of conditions, encompassing varying regimes of gene flow and strengths of ecological selection and in different biogeographical contexts including sympatry (Figure 3).

Sexual conflict has been recognized as an “engine of speciation” (Parker & Partridge, 1998; Gavrilets, 2014), but virtually all treatments of its role in population divergence focus on interlocus conflict, which can manifest itself in the form of arms races between male and female mating traits (Arnqvist et al., 2000; Gavrilets, 2000; Gavrilets and Waxman, 2002). By contrast, intralocus conflict is recognized as a contributing factor in the evolution of postzygotic isolation (see, e.g., Michalak & Noor, 2003), but its potential to enable or impede adaptive divergence is not broadly appreciated (Bonduriansky & Chenoweth, 2009). Here, we show that the resolution of intralocus sexual conflict enables a population to specialize into two alternative niches. This process bears similarity to how the evolution of a neutral female mating preference can trigger a sexually dimorphic trait to shift from one fitness peak to another, a mode of ecological speciation by sexual selection that was first modeled by Lande & Kirkpatrick, (1988) (see also De Lisle & Rowe, 2015).

### Suggestions for follow-up experiments in natural populations

This study is intended as a conceptual model that integrates various sources of data for *S. frugiperda* within the framework of ecological speciation theory and that formalizes a hypothesis for their interpretation. As a case study (cf. Gavrilets et al., 2007; Gavrilets & Vose, 2007), it also provides insight into the conditions for allochronic diversification in the fall armyworm, yielding specific predictions for further research.

First, we predict that sex differences in timing must play an important role in the maintenance of allochronic variation within populations of *S. frugiperda*. Trapping experiments using live females as lures show that rice-strain males are attracted more to females of the other strain than corn-strain males (Meagher & Nagoshi, 2013), as one would expect based on the sex-specific activity profiles that emerge in the model simulations. Interestingly, this asymmetry in cross-strain attraction is opposite to the dominant direction of interstrain matings in natural populations (Nagoshi & Meagher, 2003; Prowell et al., 2004), and we recover the same pattern in our simulations, indicating that the temporal asymmetry in competition for access to mating partners between (early) corn- and (late) rice-strain males overrides the observed bias in cross-strain attraction. Yet, measurements of sex-specific differences in the (intrinsic) circadian timing of mating activity, which could serve as a direct validation of the model, are—to our knowledge—currently lacking for *S. frugiperda*.

A second prediction (following Kirkpatrick & Ravigné, 2004) is that the maintenance of polymorphism in timing has to be supported by some form of divergent selection. This acts as a feasibility constraint throughout all of our simulation scenarios: allochronic diversification cannot occur if divergent ecological selection is absent or too weak (i.e., when the selection coefficient c is close to zero; Figure 3). Divergent ecological selection on timing may result from differential survival of juveniles between the corn and rice habitat, as we assumed here, but could also be mediated by other mechanisms. For example, ecological selection might act on timing in adults or on a different trait that is maintained in linkage disequilibrium with genetic variation in timing (Dieckmann & Doebeli, 1999). Since such alternative scenarios may conceivably differ with respect to the conditions for allochronic diversification or the way sexual conflict over timing is expressed or resolved, it is important to further elucidate how timing is involved in divergent ecological adaptation.

Clarifying the ecological context of allochronic variation in *S. frugiperda* is also important in view of current debate between experts who regard the corn- and rice-strain either as different ecological host races (Dres & Mallet, 2002; Fiteni et al., 2022; Nam et al., 2024) or as allochronic strains (Hänniger et al., 2017; Schöfl et al., 2011; Groot et al., 2010; Groot, 2014; Tessnow et al., 2022). Without denying the complexity and relevance of this discussion (Nagoshi & Meagher, 2022), we here caution against contrasting ecological host races and allochronic strains as mutually exclusive alternatives *per se*. Instead, from the perspective of adaptive speciation theory, the two interpretations emphasize complementary aspects of the speciation process—ecological divergence versus the evolution of reproductive isolation—which tend to gohand-in-hand in ecological speciation (Dieckmann et al., 2004; Weissing et al., 2011; Rettelbach et al., 2013), and that may even involve the same trait (Servedio et al., 2011). Interestingly, reproductive timing could potentially be such a multi-effect trait: apart from its role in temporal isolation, it may also contribute to divergent ecological adaptation by interacting with circadian rhythms of plant photosynthetic activity that differ between corn and rice habitats, or any of their cascading effects, such as the release of plant volatiles or the activity of habitat-specific predators or parasites.

### Evolvability of timing differences within and between species

Beyond the context of the focal species, there is ample evidence that daily (or seasonal) timing of activity (and mating) are evolvable traits that can be regulated in a sex-specific manner. In most organisms, from bacteria to higher vertebrates, daily as well as seasonal timing in behaviour and physiology are regulated by the circadian system, which is driven by an oscillating feedback loop involving clock gene transcription and time-dependent protein phosphorylation (for reviews, see, e.g. Allada et al., 2001; Zhang & Kay, 2010; Hut & Beersma, 2011; Partch et al., 2014). Several mutations that alter the abundance or function of clock proteins are known to influence the spontaneous oscillation period of the circadian clock (Saini et al., 2019), which, in turn, would effect its phase of entrainment. As a result, the daily timing of rest and activity can change by several hours due to a single-point mutation that affects the period of the oscillator (Konopka & Benzer, 1971; Ralph & Menaker, 1988).

Exaggerated effects of circadian mutations can be seen in the photoperiodic response of the neuro-endocrine axis driving reproduction. For example, hamsters, homozygous for the circadian point mutation tau, have a circadian period that is 4 hr shorter than the 24 hr period of the wildtype (Ralph & Menaker, 1988); this difference causes the mutants to have fully developed testes with under 11 hr of light per 24 hr-day, whereas wild-type hamster show fully regressed testes under these conditions (Shimomura et al., 1997).

Sex differences in circadian rhythms have been found in both invertebrates (e.g., Tauber et al., 2003; Rund et al., 2012; Floessner et al., 2019) and vertebrates (Yan & Silver, 2016; van der Vinne et al., 2019), including humans (Duffy et al., 2011). Plausible mechanisms that may have mediated the evolution of these differences include sex-specific gene regulation and hemizygosity or gene-dosage effects, for example, in haplo-diploid species or for clock genes located on a sex chromosome. In fact, a large number of circadian genes are known to be sex-linked, including *period*, *sgg*, *nocte*, *pdfr*, and *fbxl4* in *Drosophila* and *period*, *timeless*, *cycle*, and *PAR domain protein 1 pdp1* in Lepidoptera.

Given the ubiquity of temporal structure in nature and the near universal manifestation and evolvability of biological rhythms across the tree of life, it is no surprise that differences in the timing of mating are a significant source of prezygotic isolation in natural populations (Taylor & Friesen, 2017). Accordingly, insights from our study are relevant for a broader range of organisms that seem to be in the incipient stages of allochronic speciation. In addition, our analysis shows the importance of matching theoretical models of speciation with empirical data, because this combination can identify the mechanistic details of mating system and the genetic architecture of speciation traits that enable or constrain diversification in natural populations.

## Methods

The model considers a population distributed over K patches, each containing a local subpopulation of N individuals that are tracked individually by means of an agent-based simulation approach. The subpopulations are connected by the migration of adults between patches. Mating occurs within each patch after migration and is followed by the production of offspring, differential juvenile survival, and local population density regulation. Generations are discrete and nonoverlapping. In the following subsections, we describe in detail the life-cycle of our baseline model, beginning at the start of the mating season. We also outline how the baseline model is modified to simulate the alternative mating systems in Figure 1. Variants of the model that investigate the interaction between allochronic divergence and other reproductive barriers are described in Supplementary Figure S2.

### Mating systems

The mating season has a duration of T nights. We assume that males and females occur at equal frequencies at the start of the mating season, but the operational sex ratio is dynamic: males and females can (temporarily or permanently) become unavailable for mating as the mating season progresses, as a result of their previous mating interactions. The mating system of the baseline model is inspired by the biology of *S. frugiperda*. Because mating can take up to several hours in this species, we assume that no individual can mate more than once in a given night, that males can mate multiple nights, and that a receptive female mates with the first available partner that she encounters during a night. Moreover, once a female has mated, she produces a batch of eggs and is unavailable for mating in all subsequent nights.

We also considered two alternative mating systems (Figure 1) that yield qualitatively different dynamics of the operational sex ratio throughout the mating season. In the first alternative mating system (referred to as “scramble competition”), mating is assumed to take negligible time. Accordingly, if a female encounters more than one male in a given night, she can mate with all of them, and these males will immediately be available again for mating with other females. At the end of the night, mated females produce a batch of eggs and are unavailable for mating in subsequent nights. The eggs are fertilized by the sperm of one of the female’s mating partners, with all mates having equal chances of fathering the offspring. The second alternative mating system (“monogamy”) is a strictly monogamous variant of the baseline mating system. Here, after the female has encountered an available male, the pair has mated and the female has deposited a batch of eggs; both the male and the female are no longer available for mating for the remainder of the mating season.

### Determining the order of mating interactions

In *S. frugiperda*, receptive females are active during the night for a limited period of time, during which they emit sex pheromones to attract male mating partners. Once in close range, the male displays a specific calling behaviour and attempts to mate with the female. Females show limited geographic variation in the composition of their sex pheromone blend, but this variation is not consistently associated with strain type and there is no evidence of differential male attraction to the different blends (Unbehend et al., 2013). Therefore, we initially consider that mating interactions within the local subpopulation of individuals that are active within a given night are constrained only by individual activity patterns. In particular, we assumed that a male and a female have a higher probability of meeting each other when they are both active at the same time. To formalize this idea, we assumed that each individual is characterized by an activity profile $A\,\left(t;\tau_{i}\right)$ specifying the activity of individual i at time t during the night, with t ranging from 0 to 1 from the start to the end of the night. The function A is parameterized by $\tau_{i}$, the circadian timing of individual i, which we considered to be an evolvable, genetically encoded trait.

The rate of encounters between a female individual i and a male individual j was taken to be proportional to the product of their activity profiles. The number of encounters between the two individuals during a night can then be modelled as a discrete random variable $E_{i\,j}$ following a Poisson distribution


$$\begin{array}{cc} E_{i\,j}∼𝒫\,\left[\lambda \,\left(\tau_{i}^{\text{♀}},\tau_{j}^{\text{♂}}\right)\right]\,\text{ with mean}\\ & \lambda \,\left(\tau_{i}^{\text{♀}},\tau_{j}^{\text{♂}}\right)=\eta \,\int_{0}^{1}A\,\left(t;\tau_{i}^{\text{♀}}\right)\,A\,\left(t;\tau_{j}^{\text{♂}}\right)\,𝑑t, \end{array}$$ (1)

Here, we use the notation $\tau^{\text{♀}}$ and $\tau^{\text{♂}}$ to distinguish circadian timing in female and male adults from each other and from juvenile timing (henceforth denoted as $\tau^{\mathrm{juv}}$). While these three traits are encoded by the same set of genes, they may be expressed differently due to sex-specific gene expression (see under “Genetics” section; in the simulations without sex-specific gene expression, $\tau^{\text{♀}}=\tau^{\text{♂}}=\tau^{\mathrm{juv}}$ for each individual).

The timing of encounters between female i and male j is also modelled as a random variable that depends on the product of the activity profiles; its probability density function $Q\,\left(t;\tau_{i}^{\text{♀}},\tau_{j}^{\text{♂}}\right)$ is given by


$$Q\,\left(t;\tau_{i}^{\text{♀}},\tau_{j}^{\text{♂}}\right)=\frac{A\,\left(t;\tau_{i}^{\text{♀}}\right)\,A\,\left(t;\tau_{j}^{\text{♂}}\right)}{\int_{0}^{1}A\,\left(u;\tau_{i}^{\text{♀}}\right)\,A\,\left(u;\tau_{j}^{\text{♂}}\right)\,𝑑u}.$$ (2)

Throughout, we parameterized the activity profiles by functions related to the beta distribution,


$$A\,\left(t;\tau \right)=\sqrt{\frac{\Gamma \,\left(a+2\right)}{\Gamma \,\left(1+a\,\tau \right)\,\Gamma \,\left(1+a\,\left(1-\tau \right)\right)}}\,t^{\frac{a\,\tau }{2}}\,{\left(1-t\right)}^{\frac{a\,\left(1-\tau \right)}{2}},$$ (3)

where Γ denotes the gamma function and a is a (nonnegative) parameter that determines the width of an individual’s activity profile (higher values result in a more narrow profile). The first factor on the right-hand side of Equation  (3), which serves as a scaling constant, has been chosen to ensure that $A\,{\left(t;\tau \right)}^{2}$ has the form of a beta distribution (for a>0, the distribution is unimodal and has a maximum at t=τ). This implies that $\int_{0}^{1}A\,{\left(t;\tau \right)}^{2}\,𝑑t=1$ for all τ, so that the parameter η in Equation  (1) may be interpreted as the rate of encounters between individuals with identical activity profiles. Accordingly, there is no inherent advantage for any timing phenotype with respect to its rate of mate encounters within a monomorphic population. Furthermore, given Equation  (3), it can be shown that $Q\,\left(t;\tau_{i},\tau_{j}\right)=A\,{\left(t;1/2\,\left(\tau_{i}+\tau_{j}\right)\right)}^{2}$, implying that $t_{i\,j}$, the time of the encounter between individual i and j, can be sampled from a beta distribution centred around the average circadian timing trait of the two individuals.

Nonrandom mating was implemented in the individual-based simulations by first drawing $E_{i\,j}$ for each pair of individuals and time points $t_{i\,j}$ for each of their encounters. Next, proceeding from the earliest to the last interaction event, the female and male participant in each encounter were assumed to mate if and only if both partners were still available for mating at the time of their encounter (see assumptions under “Mating systems” section).

### Habitat selection, juvenile survival, and population-density regulation

We assumed that corn and rice habitats were available to each subpopulation within its local patch, but at proportions that varied between patches. Females settled in their local corn or rice habitat at the start of the mating season to eventually deposit their eggs there. The settling decision was assumed to be random in our baseline model, so that females ended up in either one or the other habitat with probability equal to the local habitat frequencies $f_{k}$ and $1-f_{k}$, respectively, for the corn and the rice habitat in patch k. As a result, the total number of females that oviposited in the corn habitat of patch k was distributed following a binomial distribution with probability $f_{k}$ and sample size N/2, so that the realized fraction of females in that habitat was determined as $F_{k}=2\,ℬ\,\left[\frac{N}{2},f_{k}\right]/N$.

Circadian timing was taken to be subject to divergent ecological selection between habitat types, mediated by differential juvenile survival. Viability selection was implemented as a soft selection mechanism, resulting in a fixed number of N offspring that survived to the adult stage in each patch. For simplicity, we assumed that females produce egg batches of a large and constant size so that offspring survival and density regulation could be modelled as a weighted lottery with replacement, where offspring were sampled in proportion to their viability, as defined below. Besides local adaptation to the habitat, juvenile survival incorporated negative density dependence and survival costs of sex-specific gene expression, which acted when juveniles developed to the adult stage and started to express their timing genes in a sex-specific manner. This cost increased with the number of timing alleles in the individual’s genome that were regulated in a sex-specific manner (by *cis*-regulatory modifications, see below); we denote this number as ℓ. Taken together, the probability of survival of a juvenile with timing trait $\tau^{\mathrm{juv}}$ in patch k was proportional to:


$$S_{k}\,\left(\tau ,\ell \right)={\left(1-s_{r}\right)}^{\frac{\ell }{2\,L}}\times \left\{ \begin{array}{cc} \frac{f_{k}}{F_{k}}\,e^{-c\,{\left(\tau -\tau_{\mathrm{corn}}^{*}\right)}^{2}} & \text{in the corn habitat}\\ \frac{1-f_{k}}{1-F_{k}}\,e^{-c\,{\left(\tau -\tau_{\mathrm{rice}}^{*}\right)}^{2}} & \text{in the rice habitat} \end{array}\right.,$$ (4)

Here, $s_{r}$ quantifies the cost of sex-specific gene regulation, and 2⁢L is the number of timing alleles in the individual’s genome. Furthermore, survival is proportional to the strength of competition experienced in the corn and rice habitat (quantified, respectively, by the ratios $f_{k}/F_{k}$ and $\left(1-f_{k}\right)/\left(1-F_{k}\right)$); and to the individual’s degree of local adaptation, which is reflected by a Gaussian selection function where $\tau_{\mathrm{corn}}^{*}$ and $\tau_{\mathrm{rice}}^{*}$ represent, respectively, the optimal timing phenotype in the corn and rice habitat, and c is a parameter that quantifies the strength of ecological divergent selection. In the initial simulations without sex-specific gene expression, we computed viability as $S_{k}\,\left(\tau ,0\right)$ for each individual.

### Migration between patches

A fraction m of the adults dispersed from their natal patch to a migrant pool, from where they subsequently dispersed to a random accessible patch in the meta-population. All patches were assumed to be accessible (i.e., there was a single global migrant pool), except in the secondary contact scenario, where the meta-population was split into two isolated clusters (each consisting of K/2 patches) before the time of secondary contact.

### Genetics

Circadian timing was encoded by a set of L diploid, bi-allelic loci with free recombination between loci. Alleles interacted additively and had equal phenotypic effect sizes ±δ, chosen such that phenotypic values for τ could range from 0 to 1. Changes in allelic state by mutation occurred at rate μ per allele per generation for all of the alleles.

We implemented the potential for sex-specific regulation of the timing genes by modelling *cis*-regulatory elements that affected gene expression either in male adults or in female adults (but not both). In particular, for sex-differential expressed genes, the allelic effect (+δ or −δ) was reversed in adults of one of the two sexes, relative to the (default) value expressed in juveniles and the other sex. We considered three types of mutations that could shape the sex-specific gene expression pattern of an allele (Supplementary Table S1): (1) regulatory mutations that switch on sex-differential expression in one of the sexes; (2) regulatory mutations that switch off sex-differential expression; and (3) mutations that reverse the juvenile (default) expression state of a sex-differentially expressed gene without affecting its expression pattern in adults. Regulatory mutations increased the total rate of mutations per allele per generation to three times μ at all loci encoding circadian timing.

### Default parameter values and initial conditions

Unless indicated otherwise, parameters used in the simulations were: K=100 (#patches), N=500 (#individuals per patch), T=100 (duration of mating season), η=25.0 (encounter rate), a=50.0 (narrowness of activity profile), c=1.0 (strength of divergent ecological selection on timing), $\tau_{\mathrm{corn}}^{*}=0.0$, $\tau_{\mathrm{rice}}^{*}=1.0$ (optimal timing in corn and rice habitat), $s_{r}=0.05$ (cost of sex-specific gene expression), m=0.01 (migration rate), L=20 (#diploid gene loci per trait), and $\mu =1.0\times 10^{-5}$ (mutation rate).

In all simulations, the meta-population consisted of K/2 patches that were rich in corn habitat ($f_{k}=0.8$, for k=1⁢…⁢K/2) and K/2 patches that were rich in rice habitat ($f_{k}=0.2$, for k=K/2+1⁢…⁢K). All patches were initialized with a starting population of genetically similar individuals with initial circadian timing $\tau_{0}=0.5$, except for the secondary contact scenario. Here, the two parts of the meta-population were initialized with subpopulations that were locally adapted but slightly displaced from the optimum of their dominant habitat type ($\tau_{0}=0.1$ in patch k=1⁢…⁢K/2 and $\tau_{0}=0.9$ in patch k=K/2+1⁢…⁢K, which are close to $\tau_{\mathrm{corn}}^{*}=0.0$ and $\tau_{\mathrm{rice}}^{*}=1.0$, respectively).

## Supplementary material

Supplementary material is available online at *Evolution Letters*.

## Supplementary Material

qrae049_suppl_Supplementary_Material

## Data Availability

C^++^ code of the simulation software used in this study is available in the Dryad Digital Repository at https://doi.org/10.5061/dryad.pvmcvdnvj.

## References

[CIT0001] Allada, R., Emery, P., Takahashi, J., & Rosbash, M. (2001). Stopping time: The genetics of fly and mouse circadian clocks. Annual Review of Neuroscience, 24, 1091–1119. 10.1146/annurev.neuro.24.1.1091 11520929

[CIT0002] Arnqvist, G., Edvardsson, M., Friberg, U., & Nilsson, T. (2000). Sexual conflict promotes speciation in insects. Proceedings of the National Academy of Sciences of the United States of America, 97, 10460–10464. 10984538 10.1073/pnas.97.19.10460 PMC27046

[CIT0003] Bateman, M., Day, R., Edgington, S., Kuhlmann, U., & Cock, M. (2018). Assessment of potential biopesticide options for managing fall armyworm (*Spodoptera frugiperda*) in africa. Journal of Applied Entomology, 142, 805–819.

[CIT0004] Bonduriansky, R. & Chenoweth, S. (2009). Intralocus sexual conflict. Trends in Ecology & Evolution, 24, 280–288. 19307043 10.1016/j.tree.2008.12.005

[CIT0005] Bürger, R., Schneider, K., & Willensdorfer, M. (2006). The conditions for speciation through intraspecific competition. Evolution, 60, 2185–2206. 17236413

[CIT0006] Campbell, C., Poelstra, J., & Yoder, A. (2018). What is speciation genomics? The roles of ecology, gene flow, and genomic architecture in the formation of species. Biological Journal of the Linnean Society, 124, 561–583.

[CIT0007] Coyne, J. & Orr, H. (2004). Speciation. Oxford University Press.

[CIT0008] De Lisle, S. & Rowe, L. (2015). Independent evolution of the sexes promotes amphibian diversification. Proc. R. Soc. Lond. B, 282, 20142213. 10.1098/rspb.2014.2213 PMC4345436 25694616

[CIT0009] Dieckmann, U. & Doebeli, M. (1999). On the origin of species by sympatric speciation. Proceedings of the Royal Society B: Biological Sciences, 282, 354–357. 10.1038/22521 10432112

[CIT0010] Dieckmann, U., Doebeli, M., Metz, J., & Tautz, D., editors (2004). Adaptive Speciation. Cambridge University Press.

[CIT0011] Doebeli, M. (2005). Adaptive speciation when assortative mating is based on female preference for male marker traits. Journal of Evolutionary Biology, 18, 1587–1600. 16313470 10.1111/j.1420-9101.2005.00897.x

[CIT0012] Dopman, E., Robbins, P., & Seaman, A. (2010). Components of reproductive isolation between north american pheromone strains of the european corn borer. Evolution, 64, 881–902. 19895559 10.1111/j.1558-5646.2009.00883.x PMC2857697

[CIT0013] Dres, M. & Mallet, J. (2002). Host races in plant-feeding insects and their importance in sympatric speciation. Philosophical Transactions of the Royal Society of London B, 357, 471–492. 10.1098/rstb.2002.1059 PMC1692958 12028786

[CIT0014] Duffy, J., Cain, S., Chang, A., Phillips, A., Münch, M., Gronfier, C., … Czeisler, C. (2011). Sex difference in the near-24-hour intrinsic period of the human circadian timing system. Proceedings of the National Academy of Sciences of the United States of America, 108, 15602–15608. 21536890 10.1073/pnas.1010666108 PMC3176605

[CIT0015] Durand, K., An, H., & Nam, K. (2024). Invasive fall armyworms are corn strain. Scientific Reports, 14, 5696. 38459145 10.1038/s41598-024-56301-0 PMC10923878

[CIT0016] Ellegren, H. & Parsch, J. (2007). The evolution of sex-biased genes and sex-biased gene expression. Nature Reviews Genetics, 8, 689–698. 10.1038/nrg2167 17680007

[CIT0017] Felsenstein, J. (1981). Skepticism towards Santa Rosalia, or why are there so few kinds of animals? Evolution, 35, 124–138. 28563447 10.1111/j.1558-5646.1981.tb04864.x

[CIT0018] Fiteni, E., Durand, K., Gimenez, S., Meagher, R., Legeai, F., Kergoat, G., … Nam, K. (2022). Host-plant adaptation as a driver of incipient speciation in the fall armyworm (*Spodoptera frugiperda*). BMC Ecology and Evolution, 22, 133. 36368917 10.1186/s12862-022-02090-x PMC9652827

[CIT0019] Floessner, T., Boekelman, F., Druiven, S., de Jong, M., Rigter, P., & Beersma, D. (2019). Lifespan is unaffected by size and direction of daily phase shifts in nasonia, a hymenopteran insect with strong circadian light resetting. Journal of Insect Physiology, 117, 103896. 31194973 10.1016/j.jinsphys.2019.103896

[CIT0020] Floessner, T., & Hut, R. (2017). Basic principles underlying biological oscillations and their entrainment. In V. Kumar (Ed.), Biological timekeeping: Clocks, rhythms and behaviour (pp. 47–58). Springer.

[CIT0021] Fry, J. (2003). Multilocus models of sympatric speciation: Bush versus Rice versus Felsenstein. Evolution, 57, 1735–1746. 14503616 10.1111/j.0014-3820.2003.tb00582.x

[CIT0022] Gavrilets, S. (2000). Rapid evolution of reproductive barriers driven by sexual conflict. Nature, 403, 886–889. 10706284 10.1038/35002564

[CIT0023] Gavrilets, S. (2004). Fitness landscapes and the origin of species. Princeton University Press.

[CIT0024] Gavrilets, S. (2014). Is sexual conflict an “engine of speciation”? Cold Spring Harbor Perspectives in Biology, 6, a017723. 25395295 10.1101/cshperspect.a017723 PMC4292158

[CIT0025] Gavrilets, S., & Vose, A. (2007). Case studies and mathematical models of ecological speciation. 2. Palms on an oceanic island. Molecular Ecology, 16, 2910–2921 17614906 10.1111/j.1365-294X.2007.03304.x

[CIT0026] Gavrilets, S., Vose, A., Barluenga, M., Salzburger, W., & Meyer, A. (2007). Case studies and mathematical models of ecological speciation. 1. Cichlids in a crater lake. Molecular Ecology, 16, 2893–2909. 17614905 10.1111/j.1365-294X.2007.03305.x

[CIT0027] Gavrilets, S., & Waxman, D. (2002). Sympatric speciation by sexual conflict. Proceedings of the National Academy of Sciences of the United States of America, 99, 10533–10538. 12149438 10.1073/pnas.152011499 PMC124966

[CIT0028] Goergen, G., Kumar, P., Sankung, S., Togola, A., & Tamo, M. (2016). First report of outbreaks of the fall armyworm *Spodoptera frugiperda* (J E Smith) (Lepidoptera, Noctuidae), a new alien invasive pest in West and Central Africa. Plos One, 11, e0165632. 27788251 10.1371/journal.pone.0165632 PMC5082806

[CIT0029] Groot, A. (2014). Circadian rhythms of sexual activities in moths: A review. Frontiers in Ecology and Evolution, 2, 43.

[CIT0030] Groot, A., Marr, M., Heckel, D., & Schöfl, G. (2010). The roles and interactions of reproductive isolation mechanisms in fall armyworm (lepidoptera: Noctuidae) host strains. Ecological Entomology, 35, 105–118.

[CIT0031] Groot, A., Marr, M., Schöfl, G., Lorenz, S., Svatos, A., & Heckel, D. (2008). Host strain specific sex pheromone variation in *Spodoptera frugiperda*. Frontiers in Zoology, 5, 20. 19109878 10.1186/1742-9994-5-20 PMC2628650

[CIT0032] Hänniger, S., Dumas, P., Schöfl, G., Gebauer-Jung, S., Vogel, H., Unbehend, M., Heckel, D. G., Groot, A. T. (2017). Genetic basis of allochronic differentiation in the fall armyworm. BMC Ecology and Evolution, 17, 1–14. 10.1186/s12862-017-0911-5 PMC5339952 28264650

[CIT0033] Hau, M., Dominoni, D., Casagrande, S., Buck, C., G., W., Hazlerigg, D., … Hut, R. (2017). Timing as a sexually selected trait: The right mate at the right moment. Philosophical Transactions of the Royal Society B: Biological Sciences, 372, 20160249. 10.1098/rstb.2016.0249 PMC5647276 28993493

[CIT0034] Hauber, M., & Sherman, P. (2001). Self-referent phenotype matching: Theoretical considerations and empirical evidence. Trends in Neurosciences, 24, 609–616. 11576676 10.1016/s0166-2236(00)01916-0

[CIT0035] Hruska, A. (2019). Fall armyworm (*Spodoptera frugiperda*) management by smallholders. CAB Reviews, 14, 1–11.

[CIT0036] Hut, R., & Beersma, D. (2011). Evolution of time-keeping mechanisms: Early emergence and adaptation to photoperiod. Philosophical Transactions of the Royal Society B: Biological Sciences, 366, 2141–2154. 10.1098/rstb.2010.0409 PMC3130368 21690131

[CIT0037] Juárez, M.L., Schöfl, G., Vera, M. T., Vilardi, J. C., Murúa, M. G., Willink, E., Hänniger, S., Heckel, D. G., Groot, A. T. (2019). Population structure of *Spodoptera frugiperda* maize and rice host forms in South America: Are they host strains? Entomologia Experimentalis et Applicata, 152, 182–199.

[CIT0038] Kirkpatrick, M., & Nuismer, S. (2004). Sexual selection can constrain sympatric speciation. Proceedings of the Royal Society of London. Series B, Biological Sciences, 271, 687–693. 10.1098/rspb.2003.2645 PMC1691651 15209101

[CIT0039] Kirkpatrick, M., & Ravign´e, V. (2004). Speciation by natural and sexual selection: Models and experiments. The American Naturalist, 158, S22–S35. 10.1086/338370 18707367

[CIT0040] Konopka, R., & Benzer, S. (1971). Clock mutants of Drosophila melanogaster. Proceedings of the National Academy of Sciences of the United States of America, 68, 2112-+. 5002428 10.1073/pnas.68.9.2112 PMC389363

[CIT0041] Kopp, M., Servedio, M., Mendelson, T., Safran, R., Rodríguez, R., Hauber, M.,... van Doorn, G. (2018). Mechanisms of assortative mating in speciation with gene flow: Connecting theory and empirical research. The American Naturalist, 191, 1–20. 10.1086/694889 29244561

[CIT0042] Kost, S., Heckel, D., Yoshido, A., Marec, F., & Groot, A. (2016). A Z-linked sterility locus causes sexual abstinence in hybrid females and facilitates speciation in *Spodoptera frugiperda*. Evolution, 70, 1418–1427 27149933 10.1111/evo.12940

[CIT0043] Lande, R., & Kirkpatrick, M. (1988). Ecological speciation by sexual selection. Journal of Theoretical Biology, 133, 85–98. 3226143 10.1016/s0022-5193(88)80026-2

[CIT0044] Loyd, S. (1982). Least squares quantization in PCM. IEEE *Transactions on Information Theory*, 28, 129–137.

[CIT0045] Matessi, C., Gimelfarb, A., & Gavrilets, S. (2001). Long-term build-up of reproductive isolation promoted by disruptive selection: How far does it go? Selection, 2, 41–64.

[CIT0046] Meagher, R., & Nagoshi, R. (2013). Attraction of fall armyworm males (lepidoptera: Noctuidae) to host strain females. Environmental Entomology, 42, 751–757. 23905738 10.1603/EN13007

[CIT0047] Meagher, R., Nagoshi, R., Stuhl, C., & Mitchell, E. (2004). Larval development of fall armyworm (Lepidoptera: Noctuidae) on different cover crop plants. Florida Entomologist, 87, 454–460.

[CIT0048] Michalak, P., & Noor, M. (2003). Association of misexpression with sterility in hybrids of *Drosophila simulans* and D-mauritiana. Journal of *Molecular Evolution*, 59, 277–282. 10.1007/s00239-004-2622-y 15486701

[CIT0049] Nagoshi, R., & Meagher, R. (2003). Fall armyworm FR sequences map to sex chromosomes and their distribution in the wild indicate limitations in interstrain mating. Insect Molecular Biology, 12, 453–458. 12974950 10.1046/j.1365-2583.2003.00429.x

[CIT0050] Nagoshi, R., & Meagher, R. (2022). The Spodoptera frugiperda host strains: What they are and why they matter for understanding and controlling this global agricultural pest. Journal of Economic Entomology, 115, 1729–1743. 36515110 10.1093/jee/toac050

[CIT0051] Nam, K., Nègre, N., & Saldumando Benjumea, C. (2024). Two host-plant strains in the fall armyworm. Insect Science https://doi.org/10.1111/1744-7917.13346 PMC11632296 38437152

[CIT0052] Parker, G., & Partridge, L. (1998). Sexual conflict and speciation. Philosophical Transactions of the Royal Society of London. Series B, Biological Sciences, 353, 261–274. 9533125 10.1098/rstb.1998.0208 PMC1692203

[CIT0053] Partch, C., Green, C., & Takahashi, J. (2014). Molecular architecture of the mammalian circadian clock. Trends in Cell Biology, 24,90–99. 23916625 10.1016/j.tcb.2013.07.002 PMC3946763

[CIT0054] Pashley, D. (1986). Host-associated genetic differentiation in fall armyworm (Lepidoptera, Noctuidae)—a sibling species complex. Annals of the Entomological Society of America, 79,898–904.

[CIT0055] Pashley, D. (1988). Quantitative genetics, development, and physiological adaptation in host strains of fall armyworm. Evolution, 42, 93–102. 28563847 10.1111/j.1558-5646.1988.tb04110.x

[CIT0056] Pashley, D., Hammond, A., & Hardy, T. (1992). Reproductive isolating mechanisms in fall armyworm host strains (Lepidoptera: Noctuidae). Annals of the Entomological Society of America, 85, 400–405.

[CIT0057] Pashley, D., Hardy, T., & Hammond, A. (1995). Host effects on developmental and reproductive traits in fall armyworm strains (Lepidoptera, Noctuidae). Annals of the Entomological Society of America, 88, 748–755.

[CIT0058] Prowell, D., McMichael, M., & Silvain, J. (2004). Multilocus genetic analysis of host use, intro- gression, and speciation in host strains of fall armyworm (Lepidoptera: Noctuidae). Annals of the Entomological Society of America, 97, 1034–1044.

[CIT0059] Ralph, M., & Menaker, M. (1988). A mutation of the circadian clock in golden-hamsters. Science, 241, 1225–1227. 3413487 10.1126/science.3413487

[CIT0060] Rettelbach, A., Kopp, M., Dieckmann, U., & Hermisson, J. (2013). Three modes of adaptive speciation in spatially structured populations. The American Naturalist, 182, E215–E234. 10.1086/673488 24231546

[CIT0061] Rund, S., Lee, S., Bush, R., & Duffield, G. (2012). Strain- and sex-specific differences in daily flight activity and the circadian clock of *Anopheles gambiae* mosquitoes. Journal of Insect Physiology, 58, 1609–1619. 23068991 10.1016/j.jinsphys.2012.09.016

[CIT0062] Saini, R., Jaskolski, M., & Davis, S. (2019). Circadian oscillator proteins across the kingdoms of life: Structural aspects. BMC Biology,17, 13. 30777051 10.1186/s12915-018-0623-3 PMC6378743

[CIT0063] Saldamando Benjumea, C., Estrada-Piedrahíta, K., Velásquez-Vélez, M., & Bailey, R. (2014). Assortative mating and lack of temporality between corn and rice strains of *Spodoptera frugiperda* (Lepidoptera, Noctuidae) from Central Colombia. Journal of Insect Behavior, 27, 555–566.

[CIT0064] Santos, H., Burban, C., Rousselet, J., Rossi, J.-P., Branco, M., & Kerdelhué, C. (2011). Incipient allochronic speciation in the pine processionary moth (*Thaumetopoea pityocampa*, Lepidoptera, Notodontidae). Journal of Evolutionary Biology, 24, 146–158. 20964783 10.1111/j.1420-9101.2010.02147.x

[CIT0065] Schöfl, G., Dill, A., Heckel, D., & Groot, A. (2011). Allochronic separation versus mate choice: Nonrandom patterns of mating between fall armyworm host strains. The American Natural- ist, 177, 470–485. 10.1086/658904 21460569

[CIT0066] Schöfl, G., Heckel, D., & Groot, A. (2009). Time-shifted reproductive behaviours among fall armyworm (noctuidae: Spodoptera frugiperda) host strains: Evidence for differing modes of inheritance. Journal of Evolutionary Biology, 22, 1447–1459. 19467132 10.1111/j.1420-9101.2009.01759.x

[CIT0067] Servedio, M., van Doorn, G., Kopp, M., Frame, A., & Nosil, P. (2011). Magic traits in speciation: “Magic” but not rare? Trends in Ecology & Evolution, 26, 389–397. 21592615 10.1016/j.tree.2011.04.005

[CIT0068] Shimomura, K., Nelson, D., Ihara, N., & Menaker, M. (1997). Photoperiodic time measurement in tau mutant hamsters. Journal of Biological Rhythms, 12, 423–430. 9376641 10.1177/074873049701200504

[CIT0069] Smadja, C., & Butlin, R. (2011). A framework for comparing processes of speciation in the presence of gene flow. Molecular Ecology, 20, 5123–5140. 22066935 10.1111/j.1365-294X.2011.05350.x

[CIT0070] Tauber, E., Roe, H., Hennessy, J., & Kyriacou, C. (2003). Temporal mating isolation driven a behavioral gene in Drosophila. Current Biology, 13, 140–145. 12546788 10.1016/s0960-9822(03)00004-6

[CIT0071] Taylor, R., & Friesen, V. (2017). The role of allochrony in speciation. Molecular Ecology, 26, 3330–3342. 28370658 10.1111/mec.14126

[CIT0072] ten Cate, C., & Vos, D. (1999). Sexual imprinting and evolutionary processes in birds: A reassess- ment. Advances in the Study of Behavior, 28, 1–31.

[CIT0073] Tessnow, A., Raszick, T., Porter, P., & Sword, G. (2022). Patterns of genomic and allochronic strain divergence in the fall armyworm, *Spodoptera frugiperda* (JE Smith). Ecology and Evolution, 12, e8706. 35356552 10.1002/ece3.8706 PMC8938225

[CIT0074] Unbehend, M., Hänniger, S., Meagher, R., Heckel, D., & Groot, A. (2013). Pheromonal divergence between two strains of *Spodoptera frugiperda*. Journal of Chemical Ecology, 39, 364–376. 23456344 10.1007/s10886-013-0263-6

[CIT0075] van Doorn, G. (2009). Intralocus sexual conflict, In: The Year in Evolutionary Biology 2009. Annals of the New York Academy of Sciences, 1168, 52–71. 19566703 10.1111/j.1749-6632.2009.04573.x

[CIT0076] van der Vinne, V., Tachinardi, P., Riede, S., Akkerman, J., J., S., Daan, S., & Hut, R. (2019). Maximising survival by shifting the daily timing of activity. Ecology Letters, 22, 2097–2102. 31617283 10.1111/ele.13404 PMC6899458

[CIT0077] Weissing, F., Edelaar, P., & van Doorn, G. (2011). Adaptive speciation theory: A conceptual review. Behavioral Ecology and Sociobiology, 65, 461–480. 21423338 10.1007/s00265-010-1125-7 PMC3038232

[CIT0078] Wolf, J., & Ellegren, H. (2017). Making sense of genomic islands of differentiation in light of speciation. Nature Reviews Genetics, 18, 87–100. 10.1038/nrg.2016.133 27840429

[CIT0079] Yainna, S., Tay, W. T., Durand, K., Fiteni, E., Hilliou, F., Legeai, F., Clamens, A., Gimenez, S., Asokan, R., Kalleshwaraswamy, C., Deshmukh, S., Meagher, Jr., R., Blanco, C., Silvie, P., Brevault, T., Dassou, A., Kergoat, G., Walsh, T., Gordon, K., … Nam, K. (2022). The evolutionary process of invasion in the fall armyworm (*Spodoptera frugiperda*). Scientific, Reports, 12, 21063. 36473923 10.1038/s41598-022-25529-z PMC9727104

[CIT0080] Yamamoto, S., & Sota, T. (2009). Incipient allochronic speciation by climatic disruption of the reproductive period. Proceedings of the Royal Society B: Biological Sciences, 276, 2711–2719 10.1098/rspb.2009.0349 PMC2839949 19419992

[CIT0081] Yan, L., & Silver, R. (2016). Neuroendocrine underpinnings of sex differences in circadian timing systems. The Journal of Steroid Biochemistry and Molecular Biology, 160, 118–126. 26472554 10.1016/j.jsbmb.2015.10.007 PMC4841755

[CIT0082] Zhang, E., & Kay, S. (2010). Clocks not winding down: unravelling circadian networks. Nature Reviews Molecular Cell Biology, 11, 764–776. 20966970 10.1038/nrm2995

