# Supplementary material for: Sex-specific expression of circadian rhythms enables allochronic speciation
Source: Evol Lett. 2024 Oct 8;9(1):65–76. doi: 10.1093/evlett/qrae049 (PMC11790224; doi:10.1093/evlett/qrae049)
Supplement: qrae049_suppl_Supplementary_Material [file qrae049_suppl_supplementary_material.zip › qrae049_suppl_Supplementary_Material.pdf]

Online Supplementary Information

Supplementary Figures

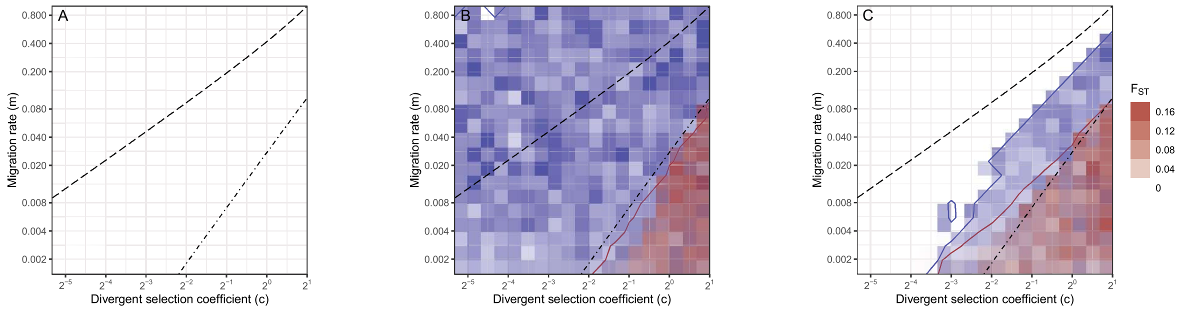

Figure S1: **Impact of the mating system on the potential for allochronic diversification.** Simulations were run for sympatric conditions (no initial divergence; red contours and shading) and a secondary contact scenario (large initial divergence and geographic isolation between subpopulations until $t = 1000$ generations; blue contours and shading), for each of the three mating systems considered in the main text: (A) mating system of *S. frugiperda* (with asymmetric contest competition between males); (B) symmetric male scramble competition; (C) monogamy. Black lines indicate analytically derived threshold curves (see Supplementary Analysis) for the emergence of polymorphism under ecological selection only (dashed), or the combined action of ecological and sexual selection (dot-dashed). After 10,000 generations of evolution, reproductive isolation was measured by quantifying F_{ST} (red and blue background shading) and the correlation in chronotype between male and female mating partners (red and blue contours indicate $r_{\mathrm{MF}} = 0.8$; $r_{\mathrm{MF}} > 0.8$ below and to the right of the contour). In panel B, F_{ST} is high and $r_{\mathrm{MF}} > 0.8$ over almost the entire parameter space in the secondary contact scenario, indicating that different chronotypes are maintained even if ecological selection is stabilizing (left and above of the dashed line). This is due to divergent sexual selection in populations with large pre-existing variation in female timing and male scramble competition; see also the discussion in the Supplementary Analysis.

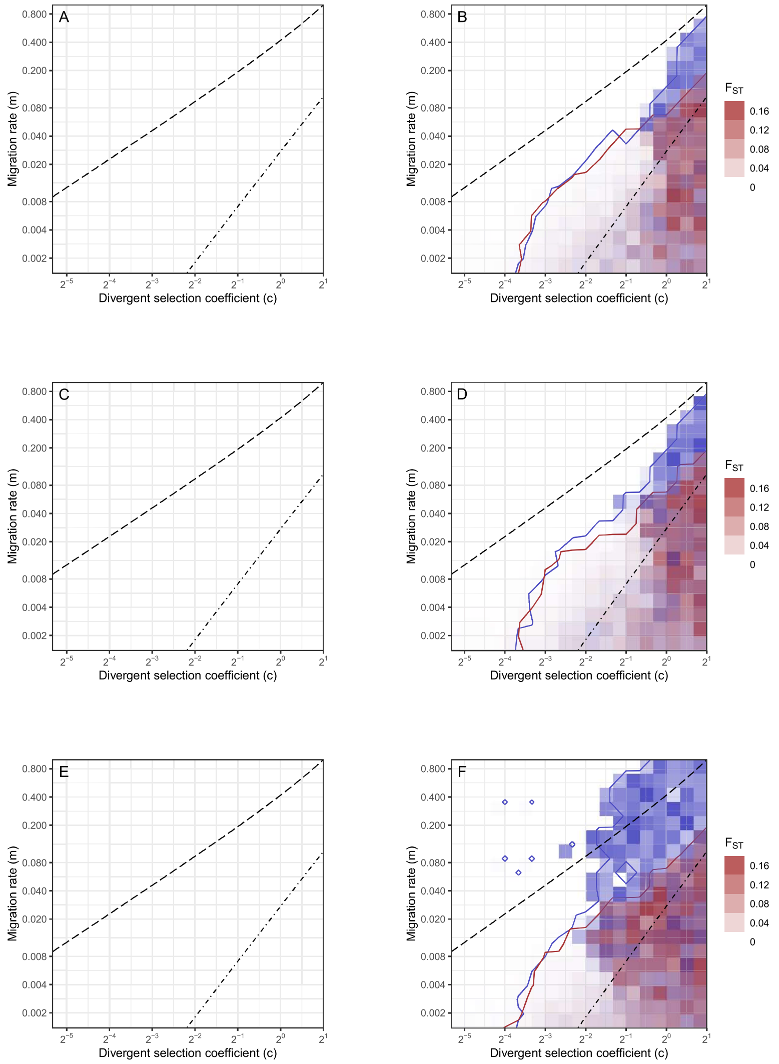

Figure S2: **Alternative reproductive barriers act in concert with allochronic differentiation, without altering its dependence on sex-specific expression.** The figure compares simulation results for the baseline model (panel A,B) with two model extensions that incorporate an additional reproductive isolating mechanism (see Supplementary Methods below): postzygotic selection against hybrids (C,D); or habitat choice, mediated by an evolving oviposition preference (E,F). For each scenario, the model was run either with (right column; B, D, F) or without sex-specific gene regulation (left column; A, C, E). Conditions for allochronic speciation are relaxed when multiple reproductive isolating mechanisms act in concert, although the effect is subtle and restricted to a secondary contact scenario (blue; sympatric conditions are shown in red, as in Fig. **3**). In all cases, sex-specific gene regulation is required for divergence to occur. Note that panel A and B replicate Fig. **S1**A, and Fig. **3**A, respectively.

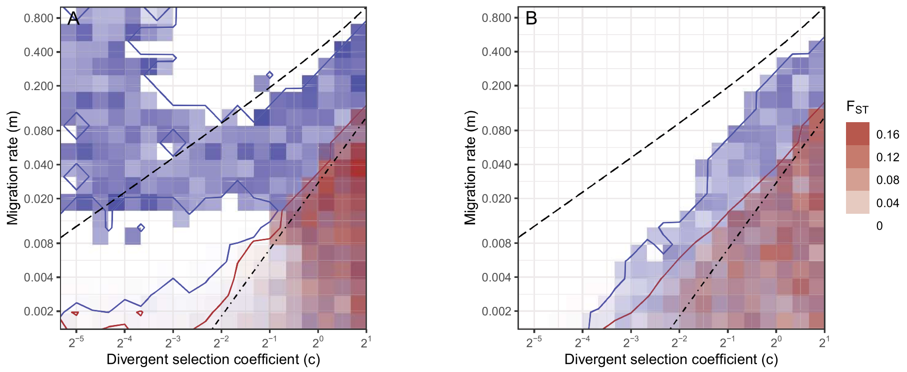

Figure S3: **No positive effects of sex-specific gene expression in alternative mating systems.** Sex-specific gene expression does not facilitate allochronic diversification in the two alternative mating systems considered in Figure **S1**. (Left) scramble competition (cf. Figure **S1**B); (Right) monogamy (cf. Figure **S1**C.)

Supplementary Table

Table S1: **Mutations at loci with sex-specific expression.** Timing loci could mutate between six different allelic states when we considered the evolution of sex-specific timing. We allowed for regulatory mutations that switch on sex-differential expression in one of the sexes (e.g., the transition from state 1 to 2, or from 6 to 4); regulatory mutations that switch off sex-differential expression (e.g., the transition from state 2 to 1, or from 4 to 6); or mutations that reverse the juvenile (default) expression state of a sex-differentially expressed gene, without affecting its expression pattern in adults (e.g., the transition from state 1 to 6, or from 3 to 5). All types of mutations are assumed to occur with equal probability, such that the total rate of mutations amounts to $3\,\mu$ per locus per generation at all loci encoding circadian timing. Allelic states 2–5 contribute to the genome-wide count of alleles with sex-differential expression (ℓ in Eq. (4)); allelic states 1 and 6 do not.

**Allelic state**	**Expression in juvenile**	**Expression in female**	**Expression in male**	**Can mutate to state:**
1	$+\delta$	$+\delta$	$+\delta$	2, 3, 6
2	$+\delta$	$+\delta$	$-\delta$	1, 3, 4
3	$+\delta$	$-\delta$	$+\delta$	1, 2, 5
4	$-\delta$	$+\delta$	$-\delta$	2, 5, 6
5	$-\delta$	$-\delta$	$+\delta$	3, 4, 6
6	$-\delta$	$-\delta$	$-\delta$	1, 4, 5

Supplementary Methods

(accompanying Supplementary Figure **S2**)

Figure **S2**CD incorporated genetic incompatibilities between $+\delta$ and $-\delta$ timing alleles, as an additional source of (postzygotic) reproductive isolation between chronotypes. Genetic incompatibilities were assumed to reduce the mate encounter rate of hybrid females (Kost et al., 2016). This was implemented by scaling the rate of mate encounters for a female by a factor corresponding to the proportion of homozygous timing loci in her genome. Given that females encountered multiple males during the mating season (depending on the mate encounter rate η), the overall strength of selection against hybrids tended to be weak, except when hybrids carried a high proportion of heterozygote loci (i.e., after populations had already diverged). Note, moreover, that this model assumes that incompatibilities occur only between divergent timing alleles that segregate at the same locus.

Figure **S2**EF show the result of simulations that allow for the evolution of active habitat choice. Here, we modelled the evolution of a genetically determined habitat trait z that mediated oviposition preference (Karpinski *et al.*, 2014). In the simulations, it was assumed that a female i in patch k selected the corn habitat with probability $H_k(z_i)$, with

$$H_k(z_i) = \frac{f_k}{f_k + (1 - f_k)\, e^{2b\,(2z_i - 1)}} \tag{1}$$

Here z_i represents the female's habitat preference ($0 \leqslant z_i \leqslant 1$), and b is a parameter that scales the strength of the habitat preference. The simulations in Figure **S2**EF were initialised with $z_0 = 0.5$ (no habitat preference), and we set $b = 5$.

Supplementary Analysis –

Divergence in generic models of phenotype matching

Our model is framed as an individual-based simulation study inspired by the biology of *S. frugiperda*, but is structured similarly to previous theoretical models of ecological divergence that have been analysed mathematically. To illustrate this relationship, we here develop an analytical treatment of our model, subject to the same simplifying assumptions as have been used in generic treatments of assortative mating as a basis for prezygotic reproductive isolation. Our aim is twofold: first, by recovering key results of earlier analyses, we will highlight common features of different models of phenotype matching; second, we will derive quantitative estimates of the strength of different components of selection during ecological speciation, to serve as a benchmark for interpreting the individual-based simulation results.

Evolutionary invasion analysis

As point of departure, we take the classical Levene (1953) model of population genetics, which analyses the conditions for the maintenance of genetic polymorphism in a population that occurs in two separate ecological niches that differ in selective conditions (i.e., the population is subject to divergent selection). Following Deakin (1966), we allow for variable levels of gene flow between the two niches and, therefore, assume that individuals mate with a partner from their own sub-population. Mating occurs after the action of viability selection and is followed by juvenile dispersal between habitats. Generations are discrete and non-overlapping.

Similar to other models of ecological speciation, we consider the evolution of a single trait, x, that is subject to divergent ecological selection and that also serves as a cue for phenotype matching (cf. Dieckmann & Doebeli, 1999). For this supplementary analysis, we do not consider sex-specific expression of x. In order to delineate conditions under which trait x is expected to diversify, we will perform an evolutionary invasion analysis and determine the conditions for evolutionary branching (Geritz *et al.*, 1998), i.e., the adaptive evolution of a polymorphism

of strategies that emerges initially in a monormorphic population, and that can subsequently diverge and be maintained over time.

For this analysis, we consider a nearly monomorphic population consisting of a large majority of resident individuals with trait value $\hat{x}$ and a low number of mutant individuals with trait value x. Let $\epsilon_k(t)$ denote the frequency of the mutant type in subpopulation k at generation t. Then, the corresponding frequency after viability selection, ϵ'_k, can be calculated as:

$$\epsilon'_k = \epsilon_k(t)\,\frac{S_k(x)}{\bar{S}_k} \approx \epsilon_k(t)\frac{S_k(x)}{S_k(\hat{x})} \tag{1}$$

Here, $S_k(x)$ represents the viability of phenotype x in subpopulation k and $\bar{S}_k = \epsilon_k(t)\,S_k(x) + (1 - \epsilon_k(t))\,S_k(\hat{x})$ denotes the mean viability in that sub-population. The approximation in the second step becomes exact in the limit of negligibly small mutant frequency ($\epsilon_k(t) \to 0$).

Earlier models of phenotype matching relied on two assumptions to accommodate non-random mating, which we will follow here. First, we take the probability of mating between two individuals to be directly proportional to $\alpha(y,z)$, a function that quantifies the relative attraction between a female with trait value y and a male with trait value z. Previous models of phenotype matching have used different assortative mating functions, but with two common features, i.e., $\frac{\partial}{\partial z}\alpha(y,z)\big|_{z=y} = 0$ and $\frac{\partial^2}{\partial z^2}\alpha(y,z)\Big|_{z=y} < 0$, reflecting the idea that females are maximally attracted to a partner that expresses a phenotype identical to their own. The second assumption is that females are the choosy sex and that males can potentially mate arbitrarily often. In fact, it is commonly assumed that females are assured of mating eventually, so that non-random mating induces no bias in the representation of female mutants among mated individuals. By contrast, the frequency of mutant males in mated pairs, ϵ''_k, will tend to deviate from their frequency after viability selection. By considering the four possible configurations of mating pairs, it follows that

$$\epsilon_k'' = \epsilon_k' \left(\underbrace{\epsilon_k' \frac{\alpha(x,x)}{\epsilon_k'\,\alpha(x,x) + (1-\epsilon_k')\,\alpha(x,\hat{x})}}_{\text{pairing success with mutant females}} + \underbrace{(1-\epsilon_k') \frac{\alpha(\hat{x},x)}{\epsilon_k'\,\alpha(\hat{x},x) + (1-\epsilon_k')\,\alpha(\hat{x},\hat{x})}}_{\text{pairing success with resident females}} \right)$$
$$\approx \epsilon_k' \frac{\alpha(\hat{x},x)}{\alpha(\hat{x},\hat{x})} \quad (2)$$

To simplify the analytical treatment, we assume that the phenotype is genetically determined by a single autosomal haploid locus. This means that the frequency of mutant individuals among the offspring produced in sub-population k is equal to $\frac{1}{2}\,\epsilon_k'' + \frac{1}{2}\,\epsilon_k'$, i.e., the average of the mutant frequency among mated males and females, respectively. A fraction m of the offspring subsequently disperse and settle at random in one of the two ecological niches. The net effect, if the subpopulations in both niches are equally large, is that a fraction $\frac{m}{2}$ of the population moves between niches per generation.

By tracing the mutant frequency through the different stages of the life cycle and combining the above results, we find that, as long as mutant individuals are rare, their frequencies in both sub-populations obey a linear recurrence relationship

$$\begin{pmatrix} \epsilon_1(t+1) \\ \epsilon_2(t+1) \end{pmatrix} = \mathbf{M} \cdot \begin{pmatrix} \epsilon_1(t) \\ \epsilon_2(t) \end{pmatrix} \quad (3)$$

where the transition matrix $\mathbf{M}$ is given by:

$$\mathbf{M} = \begin{pmatrix} 1-\frac{m}{2} & \frac{m}{2} \\ \frac{m}{2} & 1-\frac{m}{2} \end{pmatrix} \cdot \frac{1}{2}\left(1 + \frac{\alpha(\hat{x},x)}{\alpha(\hat{x},\hat{x})}\right) \cdot \begin{pmatrix} \frac{S_1(x)}{S_1(\hat{x})} & 0 \\ 0 & \frac{S_2(x)}{S_2(\hat{x})} \end{pmatrix} \quad (4)$$

The dominant eigenvalue of the matrix $\mathbf{M}$, which we will denote as λ, determines the ultimate fate of the mutant: if $|\lambda| < 1$, the frequency of the rare mutant is bound to decrease over time, such that its eventual loss from the population is inevitable; alternatively, if $|\lambda| > 1$ the mutant's frequency is expected to increase over time, so that it may potentially invade and spread to high frequency.

For this model, it is possible to obtain the dominant eigenvalue of the matrix $\mathbf{M}$ in explicit form, by solving the characteristic equation $\det(\mathbf{M} - \lambda\,\mathbf{I}) = 0$. The relevant solution is given by:

$$\lambda = \frac{1}{2}\left(1 + \frac{\alpha(\hat{x}, x)}{\alpha(\hat{x}, \hat{x})}\right) \cdot \frac{1}{2}\left(\frac{S_1(x)}{S_1(\hat{x})} + \frac{S_2(x)}{S_2(\hat{x})}\right) \cdot \left(1 - \frac{m}{2} + \sqrt{\frac{m^2}{4} + (1-m)\left(\frac{\frac{S_1(x)}{S_1(\hat{x})} - \frac{S_2(x)}{S_2(\hat{x})}}{\frac{S_1(x)}{S_1(\hat{x})} + \frac{S_2(x)}{S_2(\hat{x})}}\right)^2}\right) \tag{5}$$

The right-hand side of Eq. (5) is written as a product of three factors (separated by $\cdot$): the first factor quantifies the impact of sexual selection on the evolution of x, due to the fact that the trait underlies differential mating success in males (Kirkpatrick & Nuismer, 2004); the second and third factor reflect the fitness effects of viability selection – these terms incorporate how the costs and benefits of local adaptation are mediated by migration between subpopulations.

Analysis of the dominant eigenvalue provides information about the evolution of the phenotype, and its potential to undergo adaptive diversification (Geritz et al., 1998). The first step in this procedure is to quantify the strength and direction of directional selection by computing the selection gradient, $G(\hat{x})$. This function is given by:

$$G(\hat{x}) = \left.\frac{\partial \lambda}{\partial x}\right|_{x=\hat{x}} = \frac{1}{2}\left(\frac{S_1'(\hat{x})}{S_1(\hat{x})} + \frac{S_2'(\hat{x})}{S_2(\hat{x})}\right) \tag{6}$$

Note that the selection gradient does not depend on the mate choice function, indicating that phenotype matching does not contribute to directional selection. This is due to the assumption that $\left.\frac{\partial}{\partial z}\alpha(y, z)\right|_{z=y} = 0$, and is consistent with findings of other generic models of assortative mating (but not with our baseline individual-based simulation model, see the following section and discussion in the main text).

Potential endpoints of evolution can be found by identifying singular strategies x^* for which $G(x^*) = 0$. Such strategies are convergence-stable (i.e., attainable by a sequence of small mutational steps and adaptive trait-substitution events) if the selection gradient points towards the singular strategy in the vicinity of x^*, i.e., if

$$\left.\frac{\partial G(\hat{x})}{\partial \hat{x}}\right|_{\hat{x}=x^*} = \frac{1}{2}\left(\frac{S_1''(x^*)}{S_1(x^*)} + 2\,\frac{S_1'(x^*)}{S_1(x^*)}\frac{S_2'(x^*)}{S_2(x^*)} + \frac{S_2''(x^*)}{S_2(x^*)}\right) < 0 \tag{7}$$

A singular strategy x^* that is convergence-stable is not necessarily evolutionarily stable as well. In particular, if x^* is positioned at a local minimum of the fitness function (along the mutant-trait di-

mension), it can be invaded by neighbouring mutant strategies. The combination of convergence-stability and invasibility (i.e., lack of evolutionary stability) creates conditions conducive to evolutionary branching, i.e., the establishment of a genetic polymorphism of strategies that will tend to diverge over time as a result of subsequent mutation and trait-substitution steps. We find that the condition for x^* to be an invasible strategy evaluates to:

$$\left.\frac{\partial^2 \lambda}{\partial x^2}\right|_{x=\hat{x}=x^*} = \frac{\left.\frac{\partial^2}{\partial x^2}\alpha(x^*, x)\right|_{x=x^*}}{2\,\alpha(x^*, x^*)} + \frac{1}{2}\left(\frac{S_1''(x^*)}{S_1(x^*)} + \frac{S_2''(x^*)}{S_2(x^*)}\right) + \frac{1-m}{2\,m}\left(\frac{S_1'(x^*)}{S_1(x^*)} - \frac{S_2'(x^*)}{S_2(x^*)}\right)^2 > 0 \quad (8)$$

Comparison to the individual-based simulation model

In order to apply conditions (7) and (8) to the model developed for *S. frugiperda* in the main text, we consider the phenotypic trait x to be equivalent to timing, and specify the ecological niches 1 and 2 in the same way as in the individual-based simulations: both contain corn and rice habitat, but in different proportions. In particular, we will consider a symmetric viability selection regime:

$$S_k(x) = \begin{cases} f\,e^{-c(x-\tau_{\text{opt}})^2} + (1-f)\,e^{-c(x-(1-\tau_{\text{opt}}))^2} & \text{in niche } k=1 \\ f\,e^{-c(x-(1-\tau_{\text{opt}}))^2} + (1-f)\,e^{-c(x-\tau_{\text{opt}})^2} & \text{in niche } k=2 \end{cases}, \quad (9)$$

which is consistent with Eq. 4 of the Methods for $\tau_{\text{opt}} = \tau^*_{\text{corn}} = 1 - \tau^*_{\text{rice}} = 0$, and $f = 0.8$ (80% corn habitat in niche 1, 20% corn habitat in niche 2), the default parameter values used in the simulations.

The analytical model relies on a phenomenological description of phenotype matching that does not explicitly track the dynamics of mate availability, unlike the individual-based simulation model. Therefore, the analytical treatment cannot match the mating process of the simulations exactly. The best possible approximation is to take:

$$\alpha(y, z) = \int_0^1 A(u; y)\,A(u; z)\,du \quad (10)$$

with activity profiles $A(u; x)$ defined as in Eq. (3) of the Methods. After solving the integral, we find that $\alpha(y, z)$ can be written out in terms of Gamma functions, as follows: $\alpha(y, z) =$

$F(\frac{y+z}{2})/\sqrt{F(y)\,F(z)}$, with $F(x) = \Gamma(1 + a\,x)\,\Gamma(1 + a\,(1-x))/\Gamma(2+a)$. From here, we verify that definition (10) satisfies the assumption that $\frac{\partial}{\partial z}\alpha(y,z)\big|_{z=y} = 0$ and $\frac{\partial^2}{\partial z^2}\alpha(y,z)\Big|_{z=y} < 0$, similar to assortative mating functions used in other models of phenotype matching. The width of the activity profile, determined by parameter a, is expected to control the strength of positive assortative mating. Contourplots of the function $\alpha(y,z)$ in Fig. **S4** confirm this intuition, and show that females are more likely to select a partner that expresses a phenotype similar to their own when individuals have a more narrow activity profile.

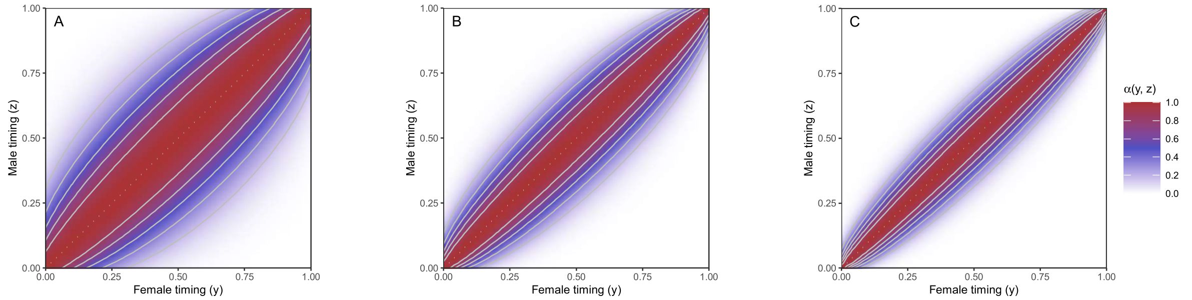

Figure S4: **Contourplots of the function** $\alpha(y,z)$. Colour scale and contours indicate the relative attraction $\alpha(y,z)$ between a female with timing trait $\tau = y$ and a male with timing trait $\tau = z$, for three different values of the parameter a that determines the width of an individual's activity profile. (A) $a = 20$, like in Fig. **3**B; (B) $a = 50$ (default parameter value); (C) $a = 100$, like in Fig. **3**C.

After substituting the definitions for S_k (Eq. 9) and $\alpha(y,z)$ (Eqs. 10, 3) into the generic fitness function (5) and following the subsequent steps in the analysis, we find that $G(x^*) = 0$ is satisfied at $x^* = \frac{1}{2}$, and that conditions (7) and (8) reduce to

$$\frac{\partial G(\hat{x})}{\partial \hat{x}}\bigg|_{\hat{x}=x^*=\frac{1}{2}} = -2\,c + 4\,c^2\,(1-2\,\tau_{\text{opt}})^2\,f\,(1-f) < 0 \tag{11}$$

$$\frac{\partial^2 \lambda}{\partial x^2}\bigg|_{x=\hat{x}=x^*=\frac{1}{2}} = -a^2\,\frac{d^2}{da^2}\ln\Gamma(1+\tfrac{a}{2}) - 2\,c + 4\,c^2\,(1-2\,\tau_{\text{opt}})^2\left(f\,(1-f) + \frac{2-m}{m}(f-\tfrac{1}{2})^2\right) > 0 \tag{12}$$

Here, $\ln \Gamma$ is the natural logarithm of the gamma function, i.e., $\ln \Gamma(1 + \frac{a}{2}) = \ln(\Gamma(1 + \frac{a}{2}))$, which has a positive second derivative for all values of a.

In order for condition (11) and (12) to hold, the habitat-specific optima for timing must be sufficiently different (requiring τ_{opt} to be sufficiently far from $\frac{1}{2}$); patches must be sufficiently different (e.g., f must be close to 0 or to 1); and gene flow must be sufficiently low (small m); finally, if both habitats are present in both patches ($0 < f < 1$), ecological selection must not be too strong, lest disruptive selection within patches undermines the maintenance of polymorphism. In addition, condition (12) is more difficult to satisfy if individuals have a narrow activity profile (large a), which is when assortative mating generates strong stabilising selection on timing. Fig. **S5** visualises these conditions for different values of the parameter a.

The general pattern visible in Fig. **S5** is that evolutionary branching depends on a combination of low gene flow and strong selection, and that the conditions for diversification become more stringent when the specificity of phenotype matching is higher (larger a; cf. Fig. **S4**). This is consistent with the fact that assortative mating generates stabilising selection on the cue used in phenotype matching in a population with low genetic variation. The individual-based simulation results in Fig. **S1**BC indicate that this stabilising effect of assortative mating is particularly relevant for divergence in sympatry. For both mating systems (scramble competition and monogamy), the conditions for allochronic divergence in sympatry are closely delineated by the (dot-dashed) boundary line derived from the mathematical analysis. Allochronic variation is maintained under a broader range of ecological conditions in a secondary-contact scenario. For the monogamous mating system (Fig. **S1**C), these conditions fall within the range of ecological conditions permissive for branching (delineated by the long-dashed boundary line), but for a mating system with scramble competition (Fig. **S1**B), variation in timing is maintained also beyond this range. In fact, in this mating system, males evolve a compromise between matching with the most abundant female type and avoiding (indirect) mate competition. At the time of secondary contact, when large, pre-existing variation in female timing is present, sexual selection can therefore become disruptive and induce males to specialize on a subpopulation of females

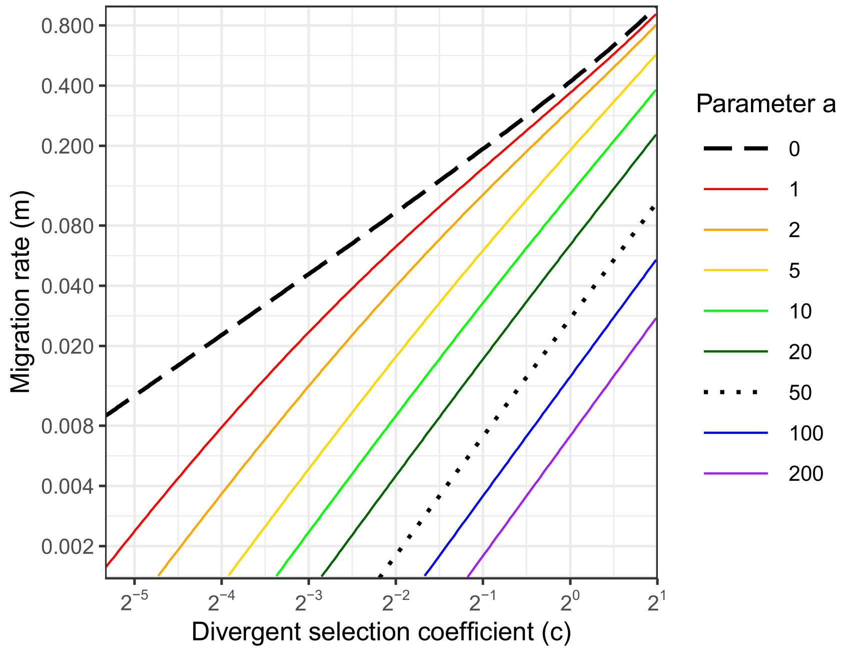

Figure S5: **Thresholds for evolutionary branching.** Inequalities (11) and (12) were evaluated for different values of the parameter a (see legend), giving rise to nine boundary lines; each line separates combinations of the key ecological parameters c (governing the strength of stabilising viability selection) and m (quantifying the level of gene flow between niches) that allow for evolutionary branching (below and to the right of the boundary line) from parameter combinations that violate inequality (11) and/or (12) (above and to the left of the boundary line). The lines that are highlighted by a different line style are used as reference lines for benchmarking the conditions for divergence in the individual-based simulations: the boundary line for $a = 50$ delineates conditions for branching in a sympatric population for the default value of a, and is shown as dot-dashed reference line in other figures, except Figure **3**BC (where it is recomputed for the alternative values of a used there); the line for $a = 0$ (long-dashed) delineates ecological conditions permissive for branching disregarding all sources of sexual selection.

in order to avoid competition with others. Polymorphism in timing can then be maintained also under conditions of net stabilizing ecological selection.

Supplementary references

Deakin, M.A.B. 1966. Sufficient conditions for genetic polymorphism. *Am.Nat.* **100**: 690–692.

Dieckmann, U., and M. Doebeli. 1999. On the origin of species by sympatric speciation. *Nature* **400**: 354–357.

Geritz, S.A.H., É. Kisdi, G. Meszéna, and J.A.J. Metz. 1998. Evolutionarily singular strategies and the adaptive growth and branching of the evolutionary tree. *Evol. Ecol.* **12**: 35–57.

Karpinski, A., S. Haenniger, G. Schöfl, D.G. Heckel, and A.T. Groot. 2014. Host plant specialization in the generalist moth Heliothis virescens and the role of egg imprinting. *Evolutionary Ecology* **28**: 1075–1093.

Kirkpatrick, M., and S.L. Nuismer. 2004. Sexual selection can constrain sympatric speciation. *Proc. R. Soc. Lond. B* **271**: 687–693.

Levene, H. 1953. Genetic equilibrium when more than one niche is available. *Am. Nat.* **87**: 331–333.
