# Supplementary figures and images for: Sex-specific expression of circadian rhythms enables allochronic speciation
Source: Evol Lett. 2024 Oct 8;9(1):65–76. doi: 10.1093/evlett/qrae049 (PMC11790224; doi:10.1093/evlett/qrae049)

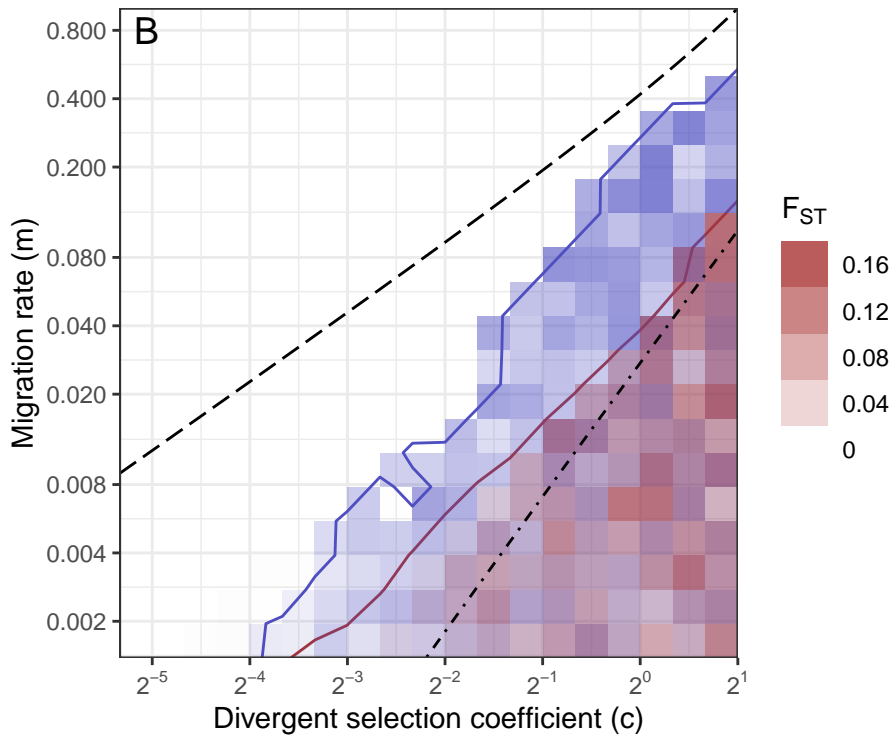

Supplement: qrae049_suppl_Supplementary_Material [file qrae049_suppl_supplementary_material.zip › qrae049_suppl_Supplementary_figS3B.pdf]

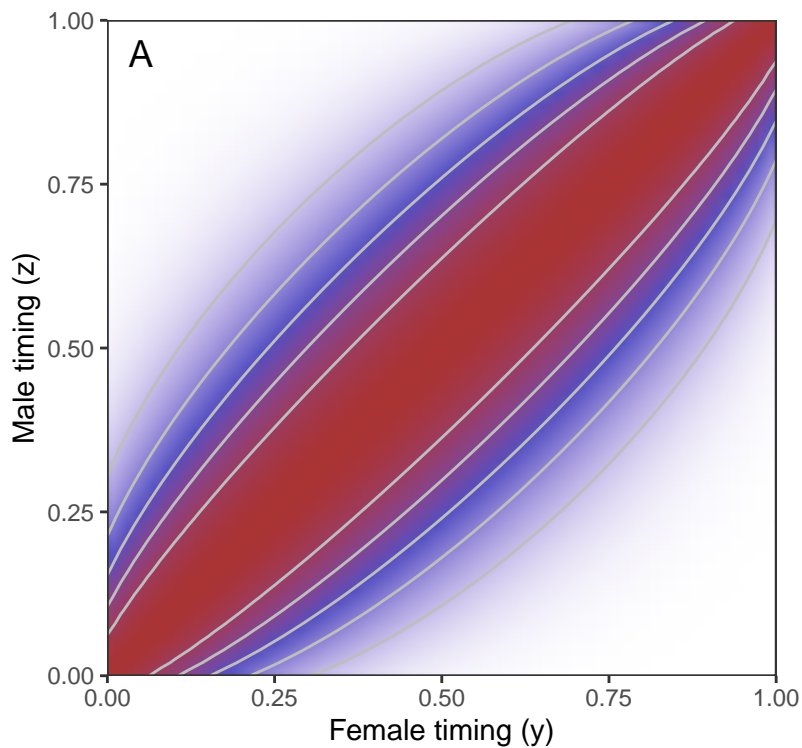

Supplement: qrae049_suppl_Supplementary_Material [file qrae049_suppl_supplementary_material.zip › qrae049_suppl_Supplementary_figS4A.pdf]

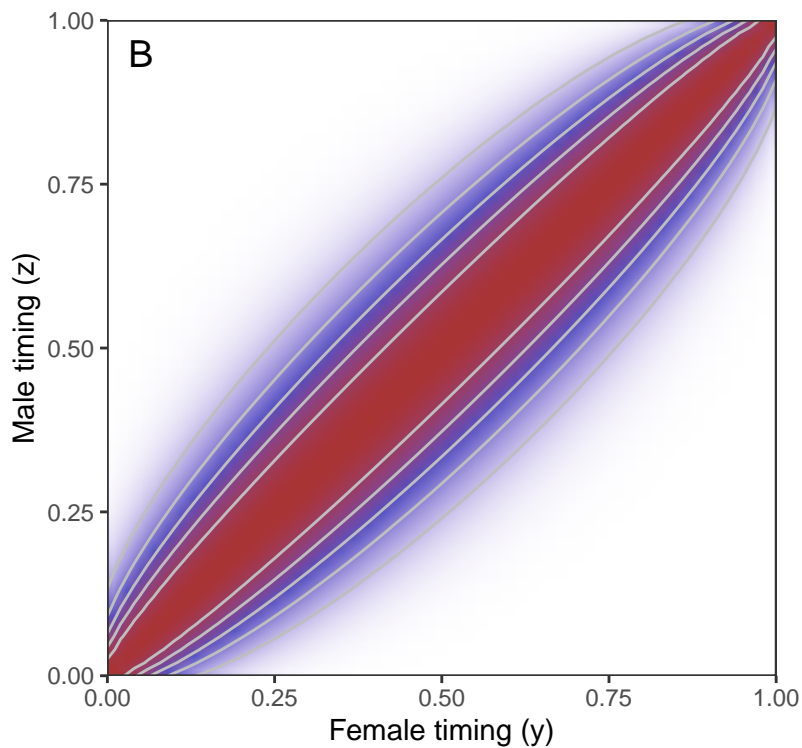

Supplement: qrae049_suppl_Supplementary_Material [file qrae049_suppl_supplementary_material.zip › qrae049_suppl_Supplementary_figS4B.pdf]

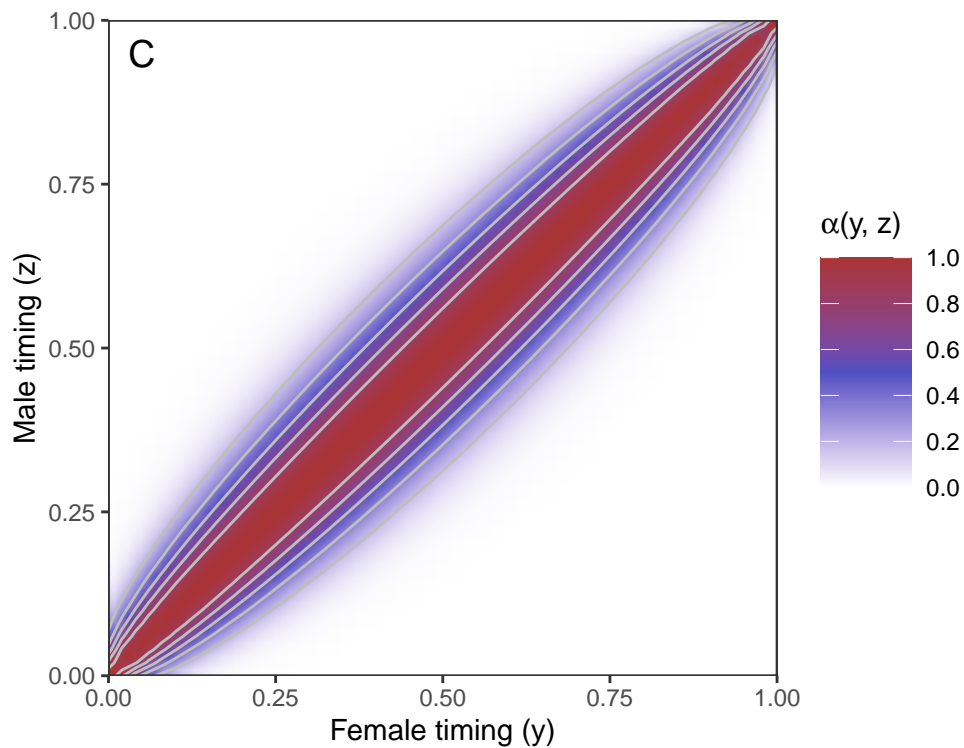

Supplement: qrae049_suppl_Supplementary_Material [file qrae049_suppl_supplementary_material.zip › qrae049_suppl_Supplementary_figS4C.pdf]

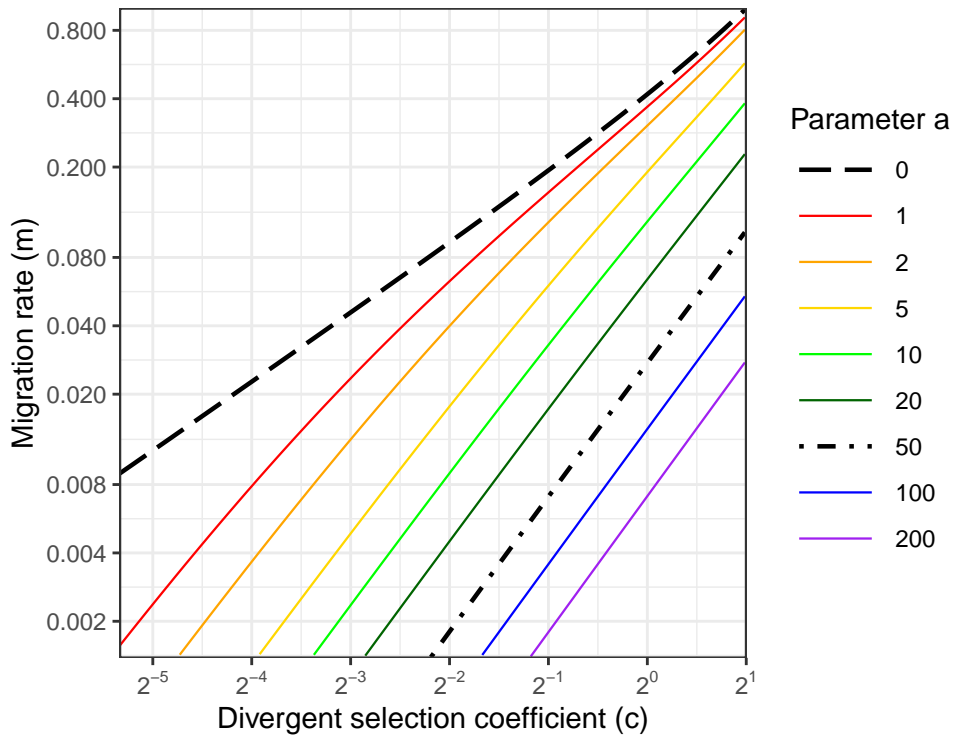

Supplement: qrae049_suppl_Supplementary_Material [file qrae049_suppl_supplementary_material.zip › qrae049_suppl_Supplementary_figS5.pdf]

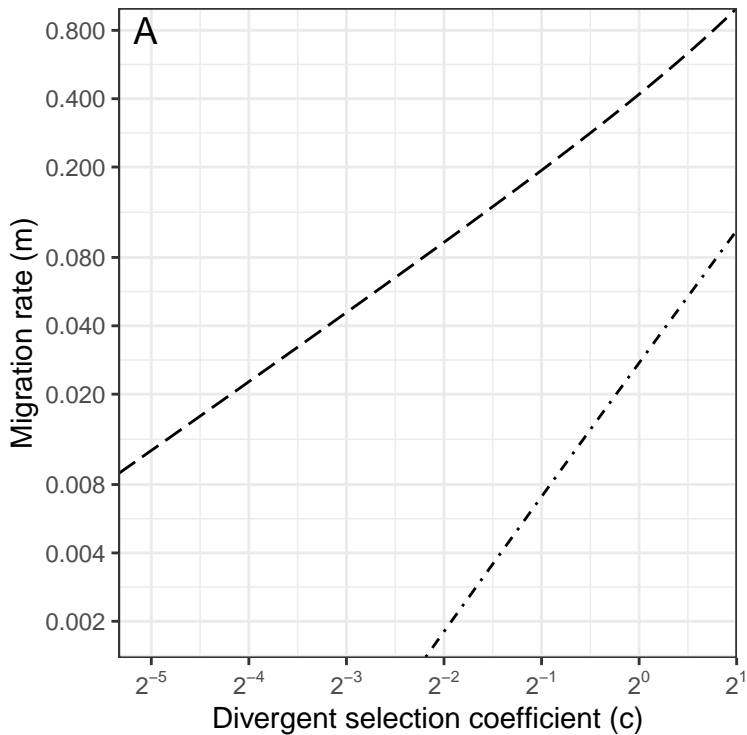

Supplement: qrae049_suppl_Supplementary_Material [file qrae049_suppl_supplementary_material.zip › qrae049_suppl_Supplementary_figS1A.pdf]

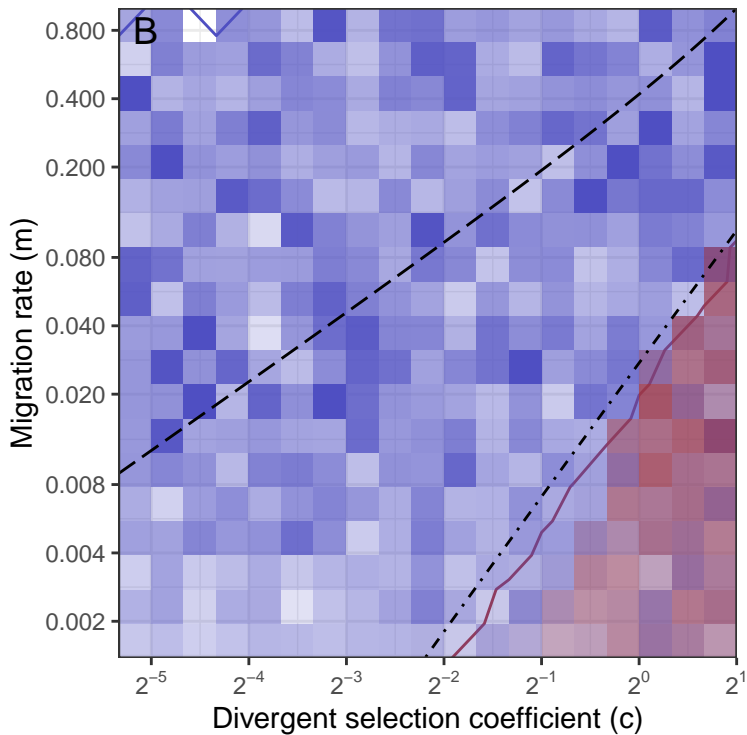

Supplement: qrae049_suppl_Supplementary_Material [file qrae049_suppl_supplementary_material.zip › qrae049_suppl_Supplementary_figS1B.pdf]

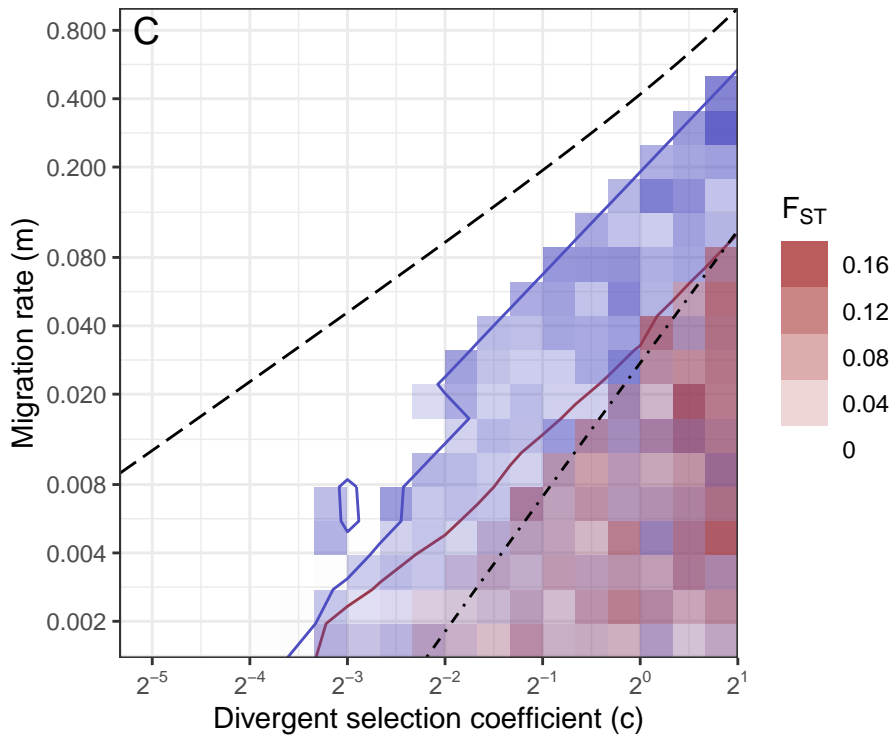

Supplement: qrae049_suppl_Supplementary_Material [file qrae049_suppl_supplementary_material.zip › qrae049_suppl_Supplementary_figS1C.pdf]

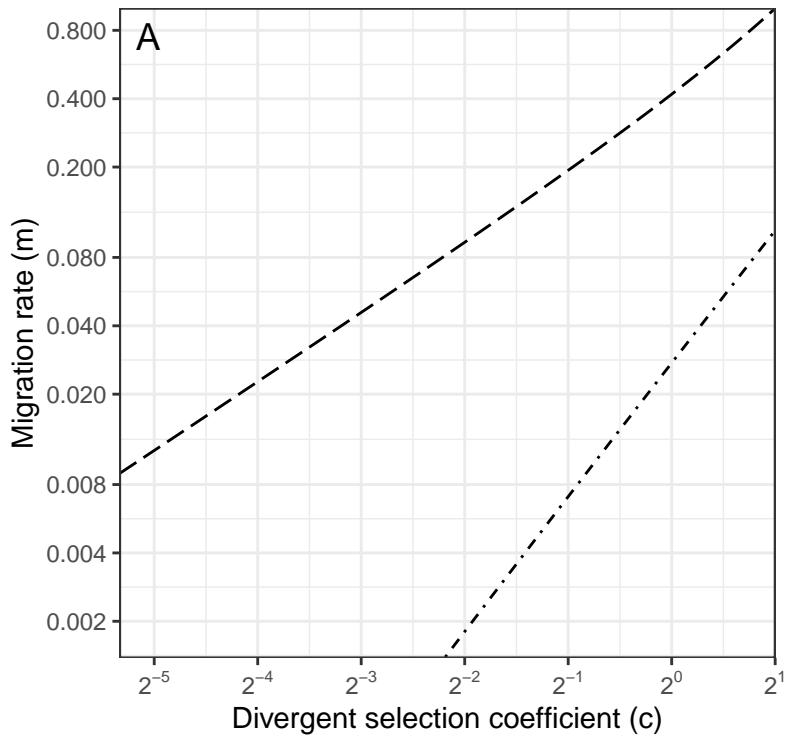

Supplement: qrae049_suppl_Supplementary_Material [file qrae049_suppl_supplementary_material.zip › qrae049_suppl_Supplementary_figS2A.pdf]

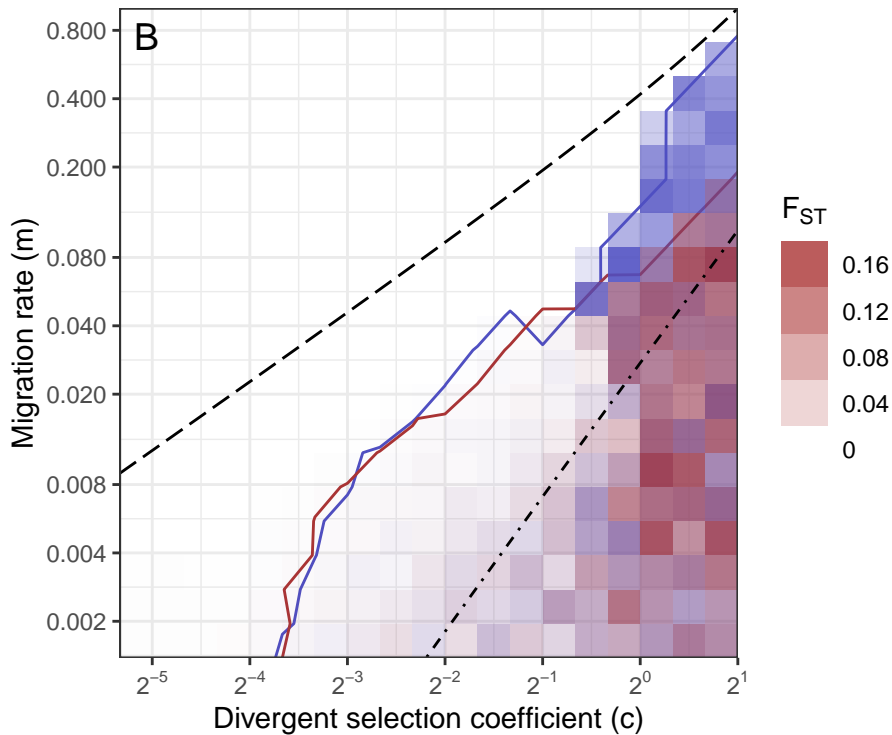

Supplement: qrae049_suppl_Supplementary_Material [file qrae049_suppl_supplementary_material.zip › qrae049_suppl_Supplementary_figS2B.pdf]

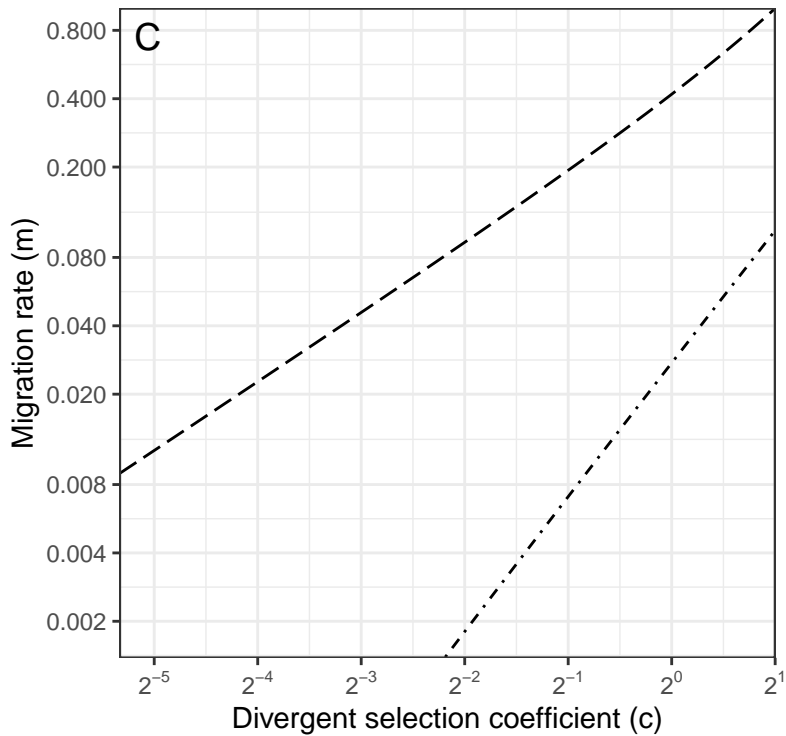

Supplement: qrae049_suppl_Supplementary_Material [file qrae049_suppl_supplementary_material.zip › qrae049_suppl_Supplementary_figS2C.pdf]

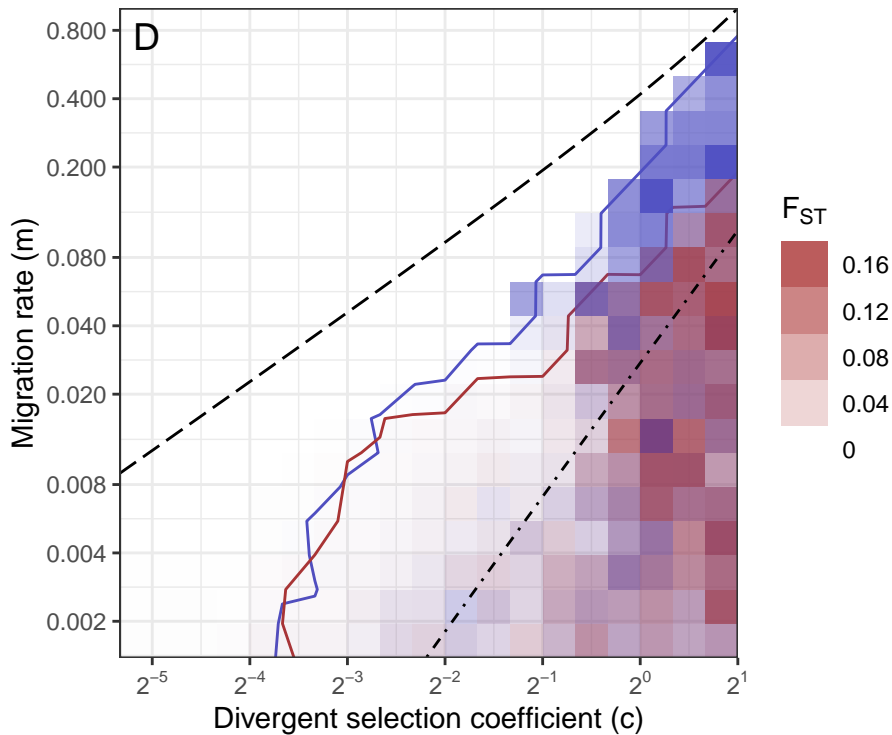

Supplement: qrae049_suppl_Supplementary_Material [file qrae049_suppl_supplementary_material.zip › qrae049_suppl_Supplementary_figS2D.pdf]

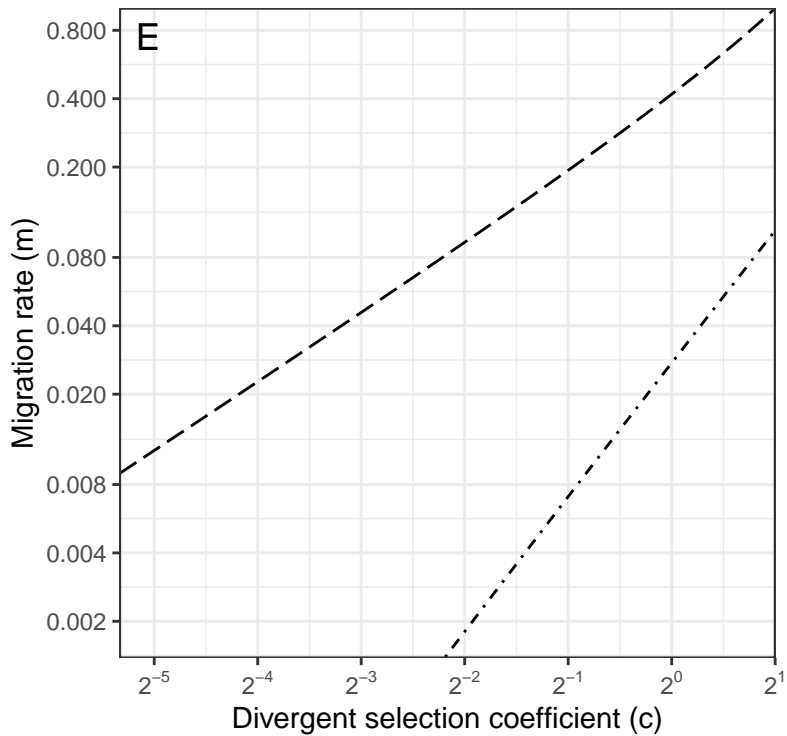

Supplement: qrae049_suppl_Supplementary_Material [file qrae049_suppl_supplementary_material.zip › qrae049_suppl_Supplementary_figS2E.pdf]

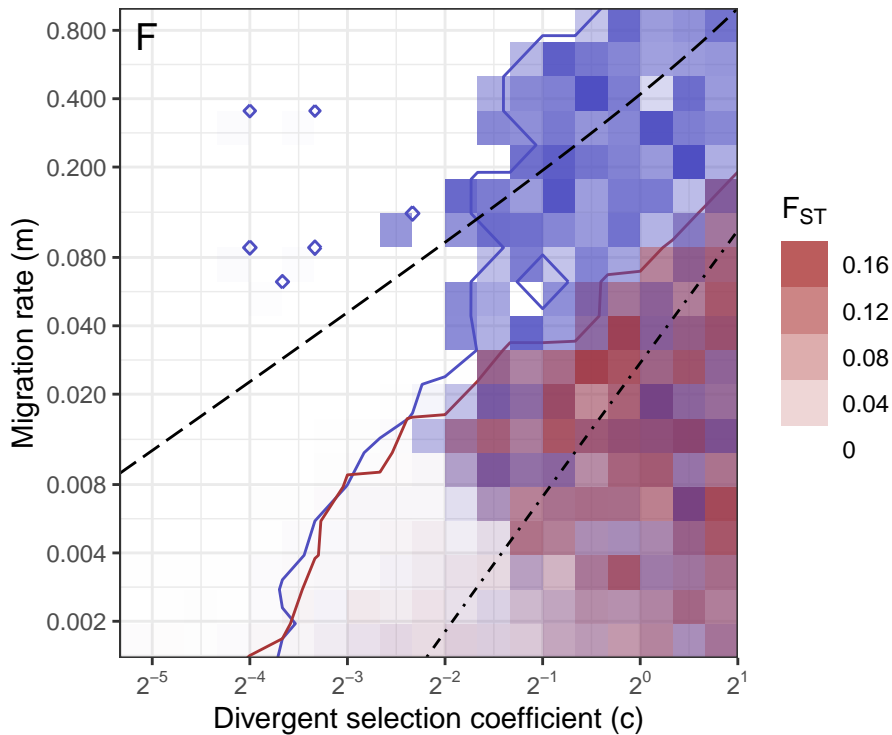

Supplement: qrae049_suppl_Supplementary_Material [file qrae049_suppl_supplementary_material.zip › qrae049_suppl_Supplementary_figS2F.pdf]

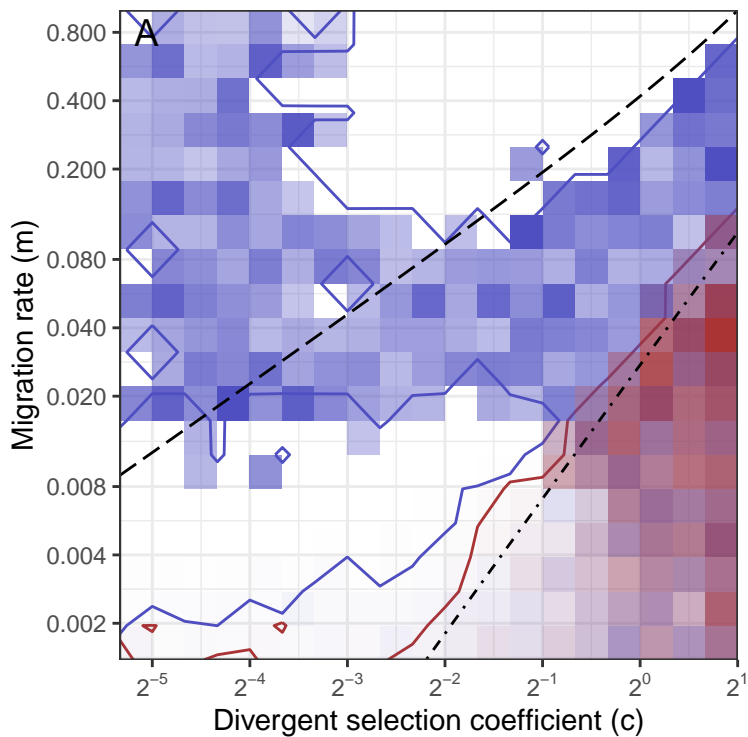

Supplement: qrae049_suppl_Supplementary_Material [file qrae049_suppl_supplementary_material.zip › qrae049_suppl_Supplementary_figS3A.pdf]
